# Transcriptomic and proteomic profiling of the anterior cingulate cortex in neuropathic pain model rats

**DOI:** 10.3389/fnmol.2023.1164426

**Published:** 2023-06-15

**Authors:** Xin-Tong Qiu, Chen Guo, Li-Tian Ma, Xin-Ning Li, Qi-Yan Zhang, Fen-Sheng Huang, Ming-Ming Zhang, Yang Bai, Guo-Biao Liang, Yun-Qing Li

**Affiliations:** ^1^Department of Anatomy, Histology and Embryology, Preclinical School of Medicine, Air Force Medical University, Xi’an, China; ^2^Department of Neurosurgery, General Hospital of Northern Theater Command, Shenyang, China; ^3^Department of Gastroenterology, Tangdu Hospital, Air Force Medical University, Xi'an, China; ^4^Institute of Neuroscience and Physiology, University of Göteborg, Göteborg, Sweden; ^5^Department of Geriatrics, Tangdu Hospital, Air Force Medical University, Xi’an, China; ^6^Department of Human Anatomy, Basic Medical College, Zunyi Medical University, Zunyi, China; ^7^Department of Anatomy, College of Basic Medicine, Dali University, Dali, China

**Keywords:** spared nerve injury, neuropathic pain (NP), anterior cingulate cortex (ACC), transcriptomics, proteomics

## Abstract

**Background:**

Neuropathic pain (NP) takes a heavy toll on individual life quality, yet gaps in its molecular characterization persist and effective therapy is lacking. This study aimed to provide comprehensive knowledge by combining transcriptomic and proteomic data of molecular correlates of NP in the anterior cingulate cortex (ACC), a cortical hub responsible for affective pain processing.

**Methods:**

The NP model was established by spared nerve injury (SNI) in Sprague–Dawley rats. RNA sequencing and proteomic data from the ACC tissue isolated from sham and SNI rats 2 weeks after surgery were integrated to compare their gene and protein expression profiles. Bioinformatic analyses were performed to figure out the functions and signaling pathways of the differentially expressed genes (DEGs) and differentially expressed proteins (DEPs) enriched in.

**Results:**

Transcriptomic analysis identified a total of 788 DEGs (with 49 genes upregulated) after SNI surgery, while proteomic analysis found 222 DEPs (with 89 proteins upregulated). While Gene Ontology and Kyoto Encyclopedia of Genes and Genomes enrichment analyses of the DEGs suggested that most of the altered genes were involved in synaptic transmission and plasticity, bioinformatics analysis of the DEPs revealed novel critical pathways associated with autophagy, mitophagy, and peroxisome. Notably, we noticed functionally important NP-related changes in the protein that occurred in the absence of corresponding changes at the level of transcription. Venn diagram analysis of the transcriptomic and proteomic data identified 10 overlapping targets, among which only three genes (XK-related protein 4, NIPA-like domain-containing 3, and homeodomain-interacting protein kinase 3) showed concordance in the directions of change and strong correlations between mRNA and protein levels.

**Conclusion:**

The present study identified novel pathways in the ACC in addition to confirming previously reported mechanisms for NP etiology, and provided novel mechanistic insights for future research on NP treatment. These findings also imply that mRNA profiling alone fails to provide a complete landscape of molecular pain in the ACC. Therefore, explorations of changes at the level of protein are necessary to understand NP processes that are not transcriptionally modulated.

## Introduction

Neuropathic pain (NP), affecting approximately 10% of the world’s population, is a common type of pathological pain resulting from a lesion or dysfunction in the somatosensory system (Finnerup et al., 2021). As a debilitating medical condition, NP takes a heavy toll on individual well-being and poses a huge challenge to both pain physicians and neurologists (Scholz et al., 2019). Current therapy predominated by CNS-acting drugs including opioids and anti-depressants fails to meet the clinical need, owing to limited efficacy and safety concerns (Cohen et al., 2021). This dilemma highlights the need for improving our understanding of the molecular basis of NP. Therefore, identifying the gene or protein expression profile in pain-associated areas is essential for understanding NP neurobiology and developing novel therapeutic strategies (Starobova et al., 2018; Gomez-Varela et al., 2019).

The anterior cingulate cortex (ACC) is a pivotal cortical area for affective pain processing (Bliss et al., 2016). Early functional data indicated that the activation of the ACC promotes behavioral sensitization and pain-related aversiveness (Lee et al., 1999; Johansen et al., 2001; Tang et al., 2005; Zhuo, 2006). With the advent of optogenetics for cell-specific manipulations, ACC glutamatergic neurons have been revealed to be effectors of nociceptive responses, while GABAergic interneurons exert opposite effects (Delevich et al., 2015; Kang et al., 2015). Further neurocircuitry studies indicated that the ACC integrates emotional pain signals from the mediodorsal and parafascicular thalamic nuclei (Meda et al., 2019; Zhu et al., 2021), and regulates emotional qualities of pain via distinct pathways differentially from behavioral hypersensitivity, with projections to the spinal dorsal horn (SDH) and striatum for behavioral hyperalgesia (Chen et al., 2018; Zhuang et al., 2021) and mesolimbic dopamine system for generating aversiveness (Gao et al., 2020). Cortical long-term potentiation (LTP) in the ACC has been esteemed as a cellular model of pathological pain (Zhuo, 2008). After nerve injury, cingulate excitatory synapses underwent long-lasting enhancements, which were attributed to enhanced presynaptic release of glutamate and postsynaptic recruitment of AMPARs and NMDARs (Xu et al., 2008). Thus, the genetic deletion of key molecules in the formation of cingulate LTP ameliorated NP in mice models (Li et al., 2010; Wang et al., 2011). Despite this strong evidence focusing on cellular mechanisms, remarkably less is known about the molecular basis underlying the neurobiology of the ACC in NP.

The past decade has witnessed a paradigm shift in the domain of pain research with the advent of multi-omics biology. This approach enables a leap forward from traditional qualitative studies of single targets to systematic investigations of multidimensional cellular networks. The ability to monitor the abundance of pain-related molecules on a genomic scale relies on high-throughput tools including RNA-sequencing and mass spectrometry-based proteomics (Nie et al., 2007; Niederberger and Geisslinger, 2008). In the pain field, analyses of mRNA and protein expression patterns have been frequently reported, but the application was limited at levels of primary sensory neurons and the SDH (Niederberger and Geisslinger, 2008; Starobova et al., 2018). In the last 2 years, emerging data have revealed transcriptomic profiles of the ACC following nerve injury (Su et al., 2021; Dai et al., 2022; Zhang et al., 2022). However, it remains hard to meet the increasing expectations of systematic pain study by investigating changes in biomolecules at a single level.

In the present study, we simultaneously utilized transcriptomic and proteomic approaches to elaborate on the complicated pathological alterations of the ACC in a rat NP model induced by spared nerve injury (SNI). These findings are of value to obtain a comprehensive view of neurobiological mechanisms underlying the role of the ACC in the modulation of NP processing and to suggest novel therapeutic targets for the treatment of NP.

## Materials and methods

### Animals and experimental design

All experimental procedures were approved by the Ethics Committee of the General Hospital of Norther Theater Command, and performed in line with the guidelines of the National Institute of Health Guide for the Care and Use of Laboratory Animals. In all, 78 adult male Sprague–Dawley rats (200–250 g) were provided by the Department of Experimental Animals of the General Hospital of Northern Theater Command. Among the animals, 36 rats were used for integrative transcriptomic and proteomic analysis, while the others were used for small RNA sequencing. The animals were housed in a reversed 12 h dark/12 h light cycle environment and provided with free access to water and food.

**Figure 1:**
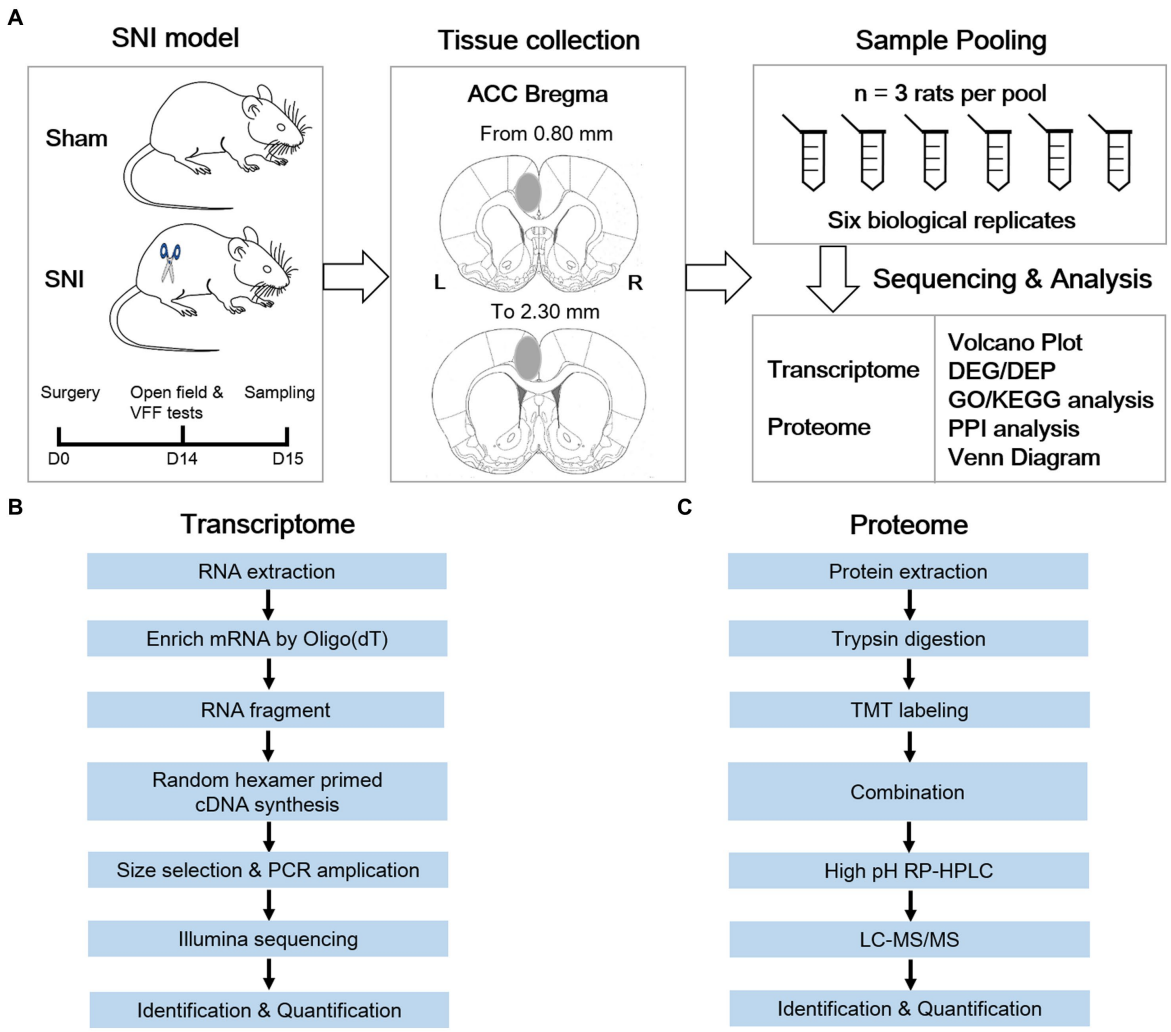
Systematic workflow of transcriptome and proteome analysis in the anterior cingulate cortex of a neuropathic pain rat model. **(A)** Schematic diagram shows the key steps of this study. **(B)** Transcriptomic workflow. **(C)** TMT proteomic workflow.

The animals were equally divided into a sham group and an SNI group. NP was elicited by SNI surgery. Two weeks after SNI surgery or sham operation, the rats underwent behavioral tests for confirming the successful establishment of the NP model. One day later, the animals were sacrificed by rapid cervical dislocation. The ACC is located adjacent to the medial frontal cortex, and these two regions have been revealed to exert different roles in pain modulation (Zhuo, 2006; Ong et al., 2019). To guarantee the accuracy of sampling, coronal brain sections (300 μm) containing the ACC were cut with a vibratome in ice-cold artificial cerebrospinal fluid (in mM: 2.6 KCl, 124 NaCl, 1 MgCl_2_, 0.5 CaCl_2_, 1.23 NaH_2_PO_4_, 26.2 NaHCO_3_, 5 kynurenic acids, 212.7 sucrose, 10 dextroses, pH 7.4), as described in previous patch-clamp experiments (Bai et al., 2019; Ren et al., 2022). The contralateral ACC was dissected out with a 15G puncher and then snap-frozen on dry ice. Afterward, RNA and protein isolation were performed on six independent pools (for integrative transcriptomic and proteomic analysis) or seven independent pools (for small RNA sequencing) of samples from the ACC, with three animals mixed as one biological pool (Figure 1; Supplementary Figure S1). To minimize the degradation of RNAs, all the materials and instruments used for sampling were treated with 0.01 M phosphate-buffered saline (PBS) containing 0.1% diethylpyrocarbonate overnight before sampling.

### SNI surgery

As described in a previous study (Ma et al., 2022), an incision was made in the hind leg to expose the biceps femoris muscle after deep anesthetization. Then, the sciatic nerve and its terminal branches, including the sural, common peroneal, and tibial nerves, were exposed. The common peroneal and tibial nerves were ligated with a 6.0 silk suture and then sectioned distal to the ligation. The muscle and skin were closed. For sham surgery, the sciatic nerve was exposed only.

### Behavioral experiments

The mechanical paw withdrawal threshold (PWT) of bilateral hind paws was calculated via the von Frey filament (VFF) test. Before testing, the animals were handled and habituated in the testing apparatus for 30 min until calming down for three consecutive days. VFFs with increasing forces from 1.4 g to 26 g were applied vertically to the lateral plantar surface of the hind paw five times, each for 5–8 s with a 5-min interval. The minimal force triggering withdrawal responses at least three times in five stimulations was considered as the PWT. Positive signs included vocalization, ipsilateral rear leg vibrating, nibbling, and withdrawal.

An open-field test was conducted to examine the locomotion as well as exploratory behaviors after SNI surgery. After acclimation in the observation room, the rats were placed in the center of the open field (100 cm × 100 cm × 60 cm). The locomotion was recorded for 15 min via a motion-tracking system (Shanghai Mobile Datum Information Technology, Shanghai, China). Anxiety-like behavior was evaluated by the total distance traveled and time spent in the center of the open field.

### RNA sequencing

Total RNA was separated with the Trizol reagent (Thermo Fisher, CA, United States). Total RNA purity and quantity were analyzed by Bioanalyzer 2,100 and RNA 6000 Nano LabChip Kit (Agilent, CA, United States). Samples with the parameters of concentration > 50 ng/uL and RIN number > 7.0 were adopted for sequencing library construction. Then, Dynabeads Oligo (dT) (Thermo Fisher) was used for the purification of mRNA with PolyA. Next, the mRNA was fragmented into small fragments with divalent cations via Magnesium RNA Fragmentation Module (NEB, MA, USA). The cleaved fragments were reverse-transcribed to create the cDNA with SuperScript™ II Reverse Transcriptase (Invitrogen, CA, United States), and then were used to synthesize U-labeled second-stranded DNAs with dUTP Solution (Thermo Fisher), RNase H (NEB), and *E. coli* DNA polymerase I (NEB). An A-base was then added to the blunt ends of each strand, preparing them for ligation to the indexed adapters. Each adapter contained a T-base overhang for ligating the adapter to the A-tailed fragmented DNA. Dual-index adapters were ligated to the fragments, and size selection was performed with AMPureXP beads. The U-labeled second-stranded DNAs were treated with the heat-labile UDG enzyme (NEB), and then the ligated products were amplified with polymerase chain reaction under the following conditions: initial denaturation at 95°C for 3 min; eight cycles of denaturation at 98°C for 15 s, annealing at 60°C for 15 s, extension at 72°C for 30 s; and final extension at 72°C for 5 min. The final cDNA libraries had a mean insert size of 300 ± 50 bp. Finally, the 2 × 150 bp paired-end sequencing was performed with the aid of an Illumina Novaseq^™^ 6,000 platform (Illumina, CA, United States).

### Transcriptomic analysis

A total of 544.78 million 2 × 150 bp paired-end reads were acquired after the RNA-seq approach. The reads were further filtered by Cutadapt^1^ (version: cutadapt-1.9). Then, the sequence quality was verified using FastQC^2^ (version: 0.11.9). All raw sequence data have been submitted to the NGDC Genome Sequence Archive (GSA) with accession number <CRA009767>. After that, a total of 79.19 Gbps of cleaned, paired-end reads were produced. The reads of all samples were aligned to the rat reference genome using the HISAT2 package^3^ (version: hisat2-2.0.4). The mapped reads of each specimen were assembled via StringTie (version: string tie-1.3.4d) with default parameters. Then, the transcriptomes from all specimens were merged to reconstruct a comprehensive transcriptome through gffcompare software (version: gffcompare-0.9.8). Then, ballgown and StringTie were utilized to perform expression abundance for mRNAs by calculating fragment per kilobase of transcript per million mapped reads (FPKM) value. Principal component analysis (PCA) was performed with the princomp function of R. The heatmaps were created via OmicShare (^1^GENE DENOVO). The analysis of differential gene expression was performed by DESeq2^4^. Those with absolute fold change (FC) ≥ 2 and false discovery rate (FDR) of less than 0.05 were considered differentially expressed genes (DEGs), which underwent subsequent enrichment analysis of Gene Ontology (GO) functions^5^ and Kyoto Encyclopedia of Genes and Genomes (KEGG) pathways^6^ with the aid of the clusterProfilter R software package.

### Small RNA sequencing and analysis

Total RNA was used as input material for RNA sample preparation. Sequencing libraries were generated using NEB Next^®^ Multiplex Small RNA Library Prep Set for Illumina^®^ (NEB E7300L). Briefly, 3′ and 5′ adaptors were ligated to the 3′ and 5′ end of small RNA, respectively. Then the first strand of cDNA was synthesized after hybridization with reverse transcription primer. The double-stranded cDNA library was generated through PCR enrichment. After purification and size selection, libraries with insertions between18 ~ 40 bp were ready for sequencing on Illumina sequencing with SE50. Subsequently, library quality was assessed on the Agilent 5400 system and quantified by qPCR (1.5 nM). The Qualified libraries were pooled and sequenced on Illumina platforms with SE50 strategy in Novogene Bioinformatics Technology Co., Ltd. (Beijing, China), according to effective library concentration and the data amount required.

Cleaning data were obtained by cleaning low-quality tags, removing adapter sequences, and filtering adaptor-ligated contaminants. The final reads were mapped to the reference sequence by Bowtie (version: 1.0.1) (Langmead et al., 2009). Clean reads were compared against small RNAs using the Rfadm database to annotate small RNS sequences. The miRBase20.0 was used to look for known miRNAs. The hairpin structures were used to predict novel miRNAs using miRDeep2 software (version: mirdeep2_0_0_5). miRNA expression levels were estimated by TPM (transcript per million) through the following criteria (Zhou et al., 2010): Normalization formula: Normalized expression = mapped read count/Total reads*1000000. Differential expression analysis of the two groups was performed using the DESeq R package (1.8.3). The *p*-values were adjusted using the Benjamini & Hochberg method. The corrected *p*-value of 0.05 was set as the threshold for significantly differential expression by default. *p-*value < 0.01 and |log_2_(fold change)| > 1 were set as the threshold for significantly differential expression by default.

Predicting the target gene of miRNA was performed by miRanda (version: miRanda-3.3a) (Enright et al., 2003) and RNAhybrid (version: RNAhybrid v2.0) (Krüger and Rehmsmeier, 2006). The targeted genes were subsequently performed for GO enrichment analysis and KEGG analyses. GOseq-based Wallenius non-central hyper-geometric distribution was implemented for GO enrichment analysis (Young et al., 2010), while KOBAS software was used for the analysis of KEGG pathways (Mao et al., 2005).

### Proteomics

After three washes in 0.01 M PBS, the tissues were ultrasonically treated for 5 min with a RIPA working solution (Beyotime, Shanghai, China). Then, the tissues were centrifuged (12,000 rpm, 4°C, 15 min) to procure the supernatant. Next, the protein solution was incubated with 5 mM dithiothreitol (Sigma-Aldrich, MO, United States) at 55°C for 20 min for reduction, followed by incubation in 15 mM iodoacetamide (Sigma-Aldrich) at room temperature (RT) for 30 min for alkylation. After digestion with filter-aided sample preparation, the solution was transferred to an Amicon filter (Millipore, MA, United States), followed by centrifugation (13,800 rpm, 25°C, 60 min) twice and the addition of 100 mM TEAB and 20% acetonitrile (Thermo Fisher). Then, the protein solution was centrifuged (13,800 rpm, 25°C, 60 min) after the addition of enzymatic buffer (100 mM TEAB, pH 7.5). At last, mass spectrometry (MS)-grade trypsin (Promega, WI, United States) was added for the first 12 h digestion, and sequencing-grade Lys-C (Wako, Japan) was added for the second 4 h digestion. After digestion, the filtrate was collected following centrifugation (13,800 rpm, 25°C, 60 min). Then concentrated to 1 mg/mL TMT reagent (Thermo Fisher) was added to 41 uL acetonitrile for re-dissolution, and this mixture was then added to the corresponding peptide segment solution and incubated (RT, 2 h) for labeling. The reaction was terminated by the addition of 5% hydroxylamine (8 uL, RT, 30 min, Sigma-Aldrich).

For each sample, 2 μg of total peptides were procured and analyzed with a nanoUPLC (EASY-nLC1200, Thermo Fisher) coupled to a Q Exactive HFX Orbitrap instrument (Thermo Fisher) with a nano-electrospray ion source. Separation was performed using a reversed-phase column (100 um ID × 15 cm, Reprosil-Pur 120C18AQ, 1.9 um, Germany). H_2_O with 0.1% formic acid (Sigma-Aldrich) and 2% acetonitrile (ANPEL Laborotory Technologies, Shanghai, China), and H_2_O with 80% ACN and 0.1% formic acid were used as mobile phases in phase A and B, respectively. Sample separation was executed at 300 nL/min flow rate with a 90 min gradient. Solvent B was set as 2–5% for 2 min, 5–22% for 68 min, 22–45% for 16 min, 45–95% for 2 min, and 95% for 2 min.

The data-dependent acquisition was processed in profile and positive mode via Orbitrap analyzer at a resolution of 120,000 (@200 m/z) and m/z range of 350–1,600 for MS1, and at a resolution of 45 k with a fixed first mass of 110 m/z for MS2. The automatic gain control target for MS1 was set to 3E6 with a max IT of 30 ms, and 1E5 for MS2 with a max IT of 96 ms. The top 20 most intense ions were fragmented by higher-energy collisional dissociation with a normalized collision energy of 32% and isolation window of 0.7 m/z. The dynamic exclusion time window was set at 45 s, and single-charged peaks and peaks with a charge exceeding six were excluded from the data-dependent acquisition.

### Proteomic analysis

The vendor’s raw MS files were processed with Proteome Discoverer (version: 2.4, Thermo Fisher) and the built-in Sequest HT search engine. MS spectra lists were searched against their species-level UniProt FASTA databases (UniProt-*Rattus norvegicus*-10,116-2021-8.fasta), with Carbamidomethyl [C], TMTpro (K), TMT pro (N-term) as the fixed modification and Oxidation (M) and Acetyl (Protein N-term) as variable modifications. The FDR was set to 0.01 for both peptide and peptide-spectrum match levels. Peptide identification was conducted with a fragment mass deviation of 0.02 Da and an initial precursor mass deviation of up to 10 ppm. The total peptide amount was used for normalization, and the razor peptide and the unique peptide were used for the quantification of proteins. The other parameters were reserved as default.

Differential protein screening followed threshold conditions of a *p*-value of less than 0.05 and 1.2-fold FC. GO and KEGG enrichment analyses were conducted on the differentially expressed proteins (DEPs) for protein function probing. The STRING database (version: 11.0)^7^ was utilized to analyze the relationships and construct the interaction network of DEPs via R language (version: 3.6.1, drawing package ggplot2).

### Data analysis

All of the normally distributed continuous variables were expressed as the mean ± standard deviation. Means were compared by unpaired *t*-tests for normally distributed data or by the Wilcoxon rank-sum (Mann–Whitney) test for non-normal data. Correlation coefficients were calculated using Pearson’s correlation test. Statistical analysis was conducted using the SPSS statistical software (IBM Corp., version: 26.0, NY, United States). A two-tailed *p*-value < 0.05 was considered significant.

## Results

### Behavioral evaluation of the rats after SNI

**Figure 2:**
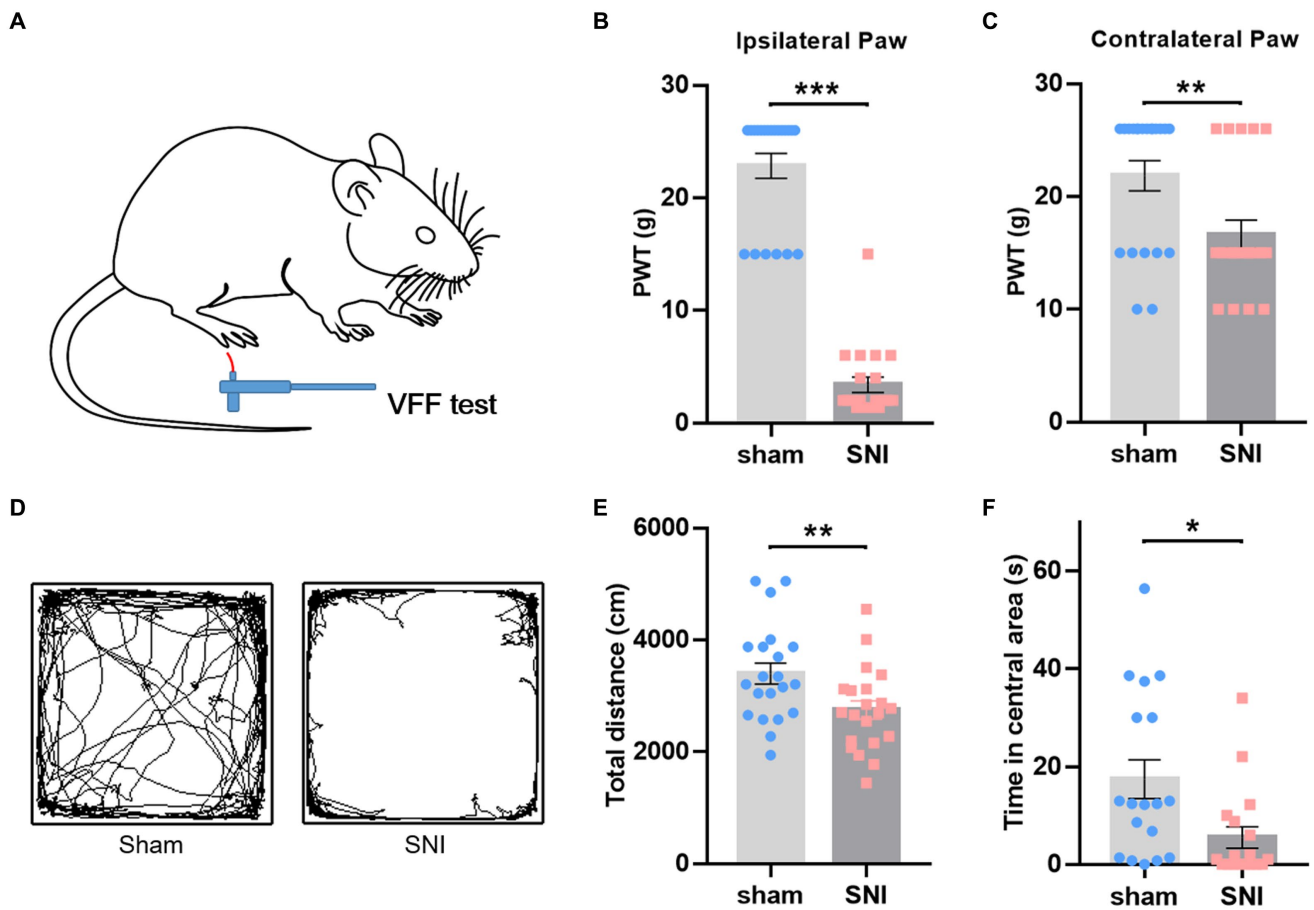
SNI-induced mechanical hyperalgesia and anxiety-like behaviors in rats. **(A)** Diagram showing the von Frey test in rats. **(B,C)** SNI-treated rats showed decreased mechanical withdrawal threshold in both ipsilateral **(B)** and contralateral **(C)** hind paws. **(D)** Representative motion trails of sham (left) and SNI (right) rats in the open field. **(E,F)** SNI-treated rats traveled less distance in the open field **(E)** and spent less time in the center of the open field **(F)** than sham rats. Unpaired *t*-test. *p < 0.05, **p < 0.01, ***p < 0.001, SNI vs. sham.

Behavioral assays were conducted 2 weeks after surgery to confirm the establishment of NP models. Compared with the sham group, SNI-treated rats displayed a significant decrease in PWT in the ipsilateral (Figures 2A,B) and contralateral hind paw (Figure 2C), indicating the existence of mirror pain. The open field test showed that SNI rats traveled less distance (Figures 2D,E) and spent less time in the central area (Figure 2F), implicating the existence of hypolocomotion and anxiety-like emotions. The behavioral performance of these rats was consistent with SNI models described in previous studies (Liu and Chen, 2014; Li Q. Y. et al., 2022). These behavioral data provided evidence of nociceptive hypersensitivity and behavioral anxiety in SNI-treated rats.

### Transcriptomic signatures in the ACC of rats following SNI

**Figure 3:**
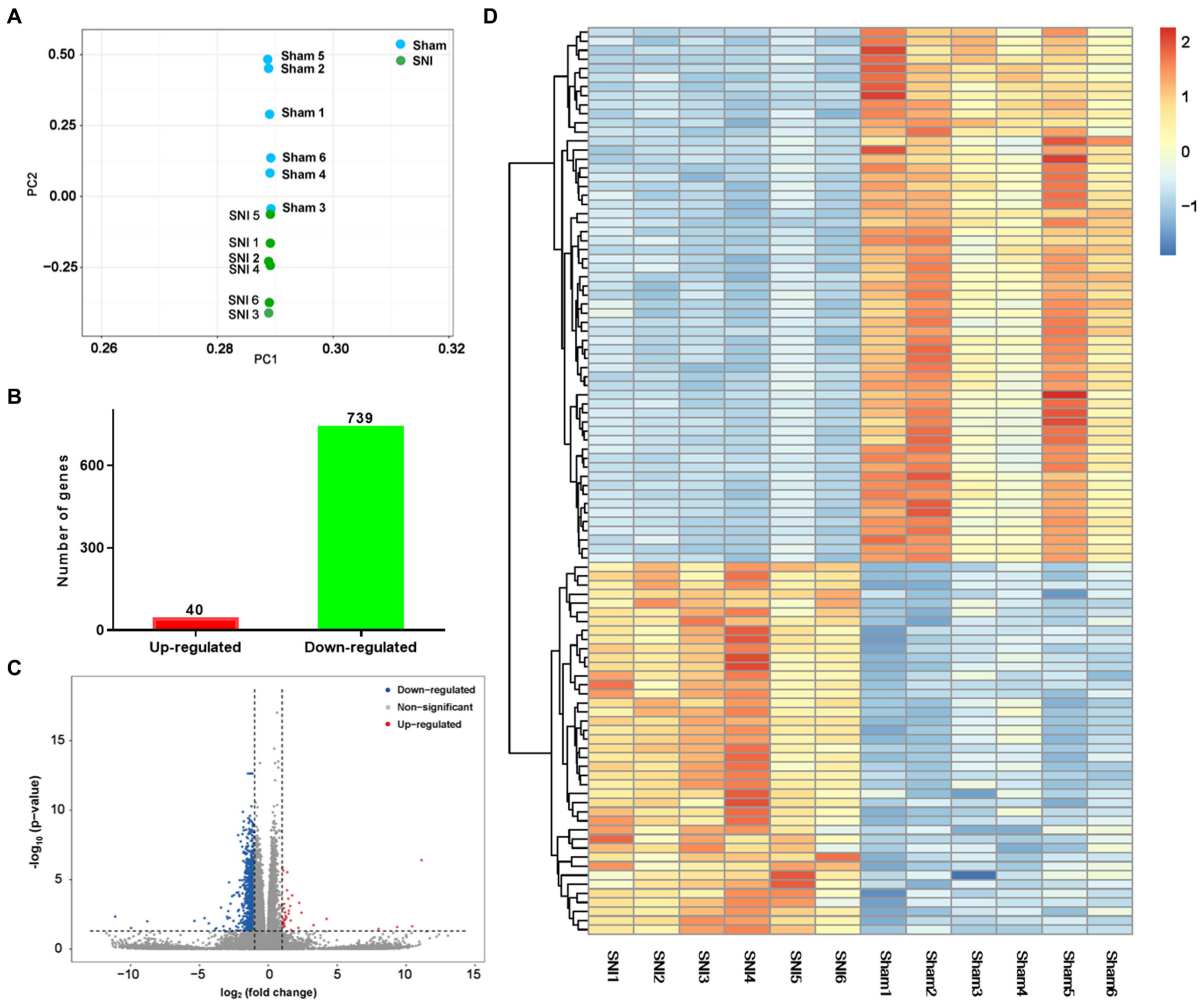
General transcriptomic outcomes. **(A)** Principal component analysis indicated a near-complete separation of genes in the sham (blue) and SNI (green) groups. **(B)** In all, 40 genes were upregulated, and 739 genes were downregulated. **(C)** Volcano plot shows differentially expressed genes. The red and blue dots indicate significantly upregulated and downregulated genes, respectively. **(D)** Hierarchical cluster analysis. Red indicates upregulation, and blue indicates downregulation. The dendrograms represent the classification of genes. The number in the color scale indicates the z-score.

The transcriptome data were acquired using the RNA-seq method from rat ACC tissue 2 weeks after SNI or sham surgery. A total of 44,000,392 clean reads were averagely generated after filtering for low-quality from 45,398,261 raw sequencing reads. The number of identified genes and their proportion and distribution to the total gene number in the database of each sample were counted and calculated (Supplementary Table S1). The distribution and density of gene expression levels are shown in Supplementary Figures S2A,B. PCA showed that the two groups of samples distinctively clustered into different categories, implying distinct gene expression patterns of NP rats (Figure 3A). The volcano plot revealed 40 upregulated DEGs and 739 downregulated DEGs in the ACC of rats 14 days after SNI (Figures 3B,C). The heatmap data showed that the top 100 DEGs according to the *q*-value could significantly distinguish these two groups (Figure 3D). Most of the DEGs (89.7%) were mRNAs (Supplementary Figure S2C). In addition, a few differentially expressed long intergenic non-coding RNAs (lincRNA, *N* = 27), microRNAs (miRNA, *N* = 5), small nucleolar RNAs (snoRNA, *N* = 7), ribosomal RNAs (rRNA, *N* = 5), and small nuclear RNA (snRNA, *N* = 3), were also identified. The top 20 upregulated and downregulated differentially expressed protein-coding RNAs are shown in Table 1, while the significant non-coding RNAs (ncRNA) are listed in Supplementary Table S2.

**Table 1:** Differentially expressed protein-coding genes in the anterior cingulate cortex after nerve injury by RNA-Seq.

Gene name	Description	Average		*p*-value	FC
		Sham	SNI		
**Downregulated genes**					
Vom1r88	Vomeronasal 1 receptor 88	0.10	0.00	0.00599	0.00
Chrm5	Cholinergic receptor, muscarinic 5	0.04	0.00	0.00153	0.00
Drd3	Dopamine receptor D3	0.22	0.01	0.00142	0.02
AC128967	Zinc finger protein 42-like	0.16	0.01	0.00791	0.07
Ccdc3	Coiled-coil domain containing 3	0.32	0.03	0.00009	0.09
Matn3	Matrilin 3	0.09	0.01	0.00426	0.10
Fshb	Follicle-stimulating hormone subunit beta	0.15	0.02	0.00060	0.13
Trappc3l	Trafficking protein particle complex 3-like	0.53	0.07	0.00088	0.13
Npy2r	Neuropeptide Y receptor Y2	0.43	0.06	0.00019	0.13
Cd300le	Cd300 molecule-like family member E	0.45	0.06	0.00073	0.14
Pag1	Phosphoprotein membrane anchor with glycosphingolipid microdomains 1	0.70	0.10	0.00004	0.15
Pcdha4	Protocadherin alpha 4	0.43	0.07	0.00001	0.16
Il13ra1	Interleukin 13 receptor, alpha 1-like	0.60	0.10	0.00140	0.17
Syt9	Synaptotagmin 9	0.95	0.19	0.00000	0.20
LOC100909830	m7GpppN-mRNA hydrolase-like	0.28	0.06	0.00019	0.22
Nos1	Nitric oxide synthase 1	1.16	0.25	0.00000	0.22
Pcdhga5	Protocadherin gamma subfamily A, 5	0.36	0.08	0.00000	0.22
Cxcl10	C-X-C motif chemokine ligand 10	0.45	0.10	0.00245	0.23
Pcdhga8	Protocadherin gamma subfamily A, 8	0.69	0.17	0.00015	0.23
Acvr1c	Activin A receptor type 1C	0.57	0.14	0.00001	0.23
**Upregulated genes**					
LOC687560	Hypothetical protein	7.4	20.5	0.00422	1384.90
Olr1104	Olfactory receptor 1104	70.0	169.8	0.00487	650.96
Trim29	Tripartite motif-containing 29	635.9	1144.1	0.00727	255.43
AABR07000740	Uncharacterized LOC103690177	62.0	108.4	0.00093	18.75
Tfap2a	Transcription factor AP-2 alpha	19.8	31.8	0.00334	9.70
Gp6	Glycoprotein 6 (platelet)	145.3	229.4	0.00027	5.29
S100a9	S100 calcium binding protein A9	891.1	1387.1	0.00004	4.71
Pcp2	PURKINJE cell protein 2	611.2	932.0	0.00587	4.55
Pou5f1	POU class 5 homeobox 1	2428.6	3697.5	0.00128	3.08
LOC100362054	mCG114696-like	91.5	134.9	0.00007	3.00
Sox15	SRY box 15	83.2	121.5	0.00000	2.55
Scp2d1	SCP2 sterol-binding domain containing 1	251.0	362.6	0.00026	2.30
LOC108348155	Paired immunoglobulin-like type 2 receptor beta-2	26.1	37.3	0.00026	2.30
Prr32	Proline rich 32	342.7	484.2	0.00415	2.25
Prss22	Serine protease 22	16.6	23.4	0.00005	2.21
Mia	MIA SH3 domain containing	222.2	313.5	0.00011	2.19
Ahsp	Alpha hemoglobin stabilizing protein	36.8	51.6	0.00490	2.17
Mpp4	Membrane palmitoylated protein 4	202.9	282.9	0.00000	2.13
Creb3l4	cAMP responsive element binding protein 3-like 4	162.4	225.8	0.00013	2.06
Tnni3	Troponin I3, cardiac type	270.6	373.9	0.00448	2.03

Twenty downregulated and upregulated differentially expressed protein-coding genes with the lowest *p*-values in the rat anterior cingulate cortex after SNI. The genes are ranked on the average of respective absolute fold change.

FC, fold change; SNI, spared nerve injury.

**Figure 4:**
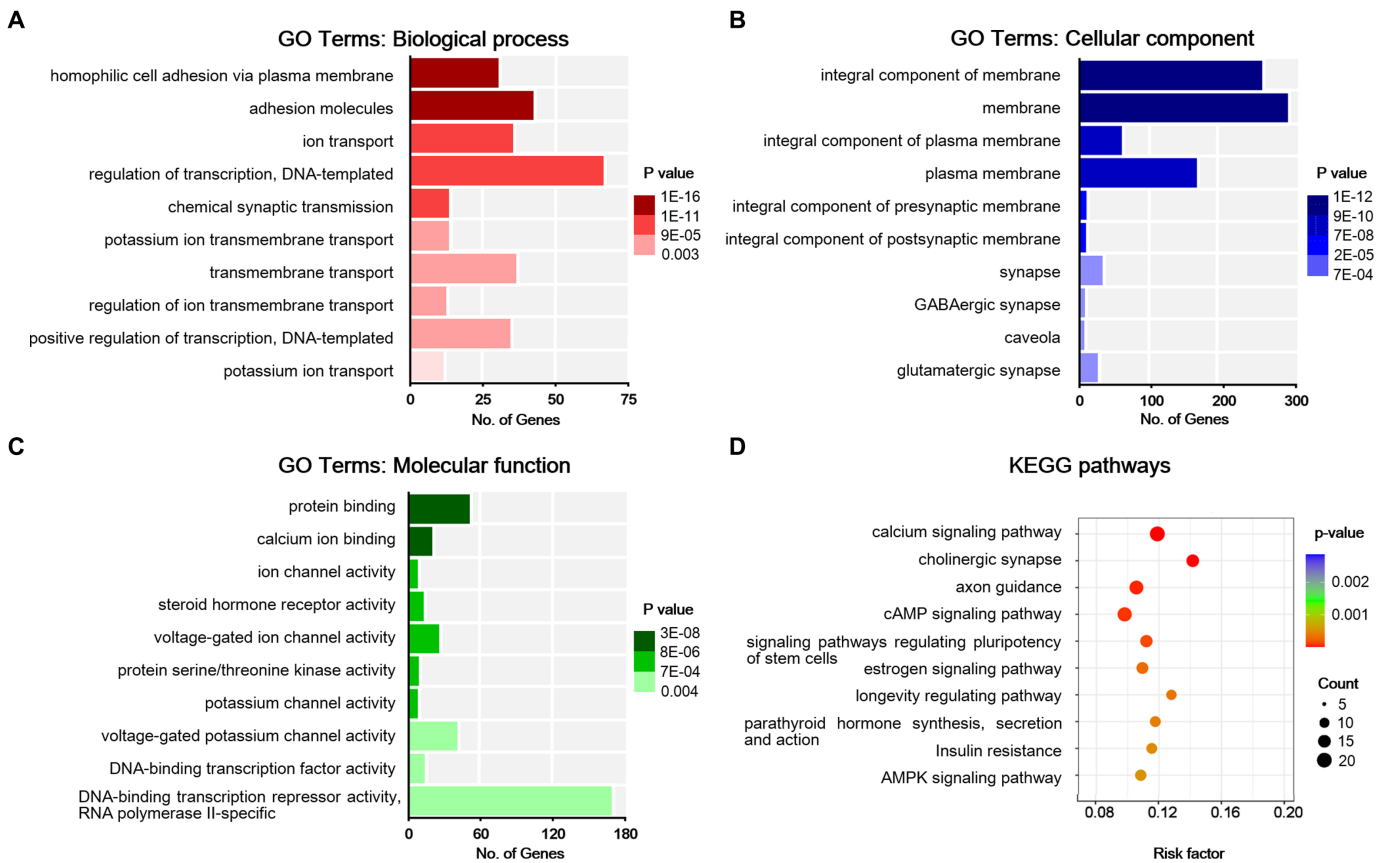
Bioinformatic analysis for differentially expressed genes in sham and NP rats. **(A)** Biological process. **(B)** Cell components. **(C)** Molecular function. Darker colors indicate higher statistical significance. **(D)** Bubble chart shows KEGG analysis of differential genes. The horizontal axis represents a rich factor (ratio of the sum of differential genes enriched in a pathway to the number of genes annotated by the pathway). Bubble size indicates the number of genes included in each pathway, and different colors indicate different *p*-values.

To understand the functions of the DEGs identified, GO analysis was performed for enrichment analysis and classifications, with the top 10 enriched items in each classification shown in Figures 4A–C. GO analysis identified biological processes (BP) associated with adhesion molecules, ion transport, homophilic cell adhesion via plasma membrane, regulation of DNA-templated transcription, regulation of ion transmembrane transport, potassium ion transport, and chemical synaptic transmission. Enriched cellular component (CC) terms associated with membrane (including caveola, presynaptic, and postsynaptic membrane) and synapse (including glutamatergic and GABAergic synapses). Moreover, identified enriched molecular functions (MF) associated with protein binding, calcium ion binding, ion channel activity, protein serine/threonine kinase activity, steroid hormone receptor activity, and potassium channel activity. We then used the DEGs for KEGG pathway enrichment (Figure 4D). The DEGs were significantly enriched in the classifications of the calcium signaling pathway, cholinergic synapse, axon guidance, cAMP signaling pathway, signaling pathways in charge of regulation of stem cell pluripotency, longevity regulating pathway, estrogen signaling pathway, parathyroid hormone synthesis, secretion and action, insulin resistance, and AMPK signaling pathway. All these suggest that peripheral nerve injury-induced synaptic activity changes in the ACC.

### MiRNAomic signatures in the ACC of rats following SNI

Differing from mRNAs, small non-coding RNAs exert functions by silencing the targeted mRNAs to regulate post-transcriptional protein accumulation and play a pivotal role in ontogenesis, tumorigenesis, and the development of various human disorders (Esteller, 2011). Interestingly, the domain of pain study is experiencing rapid growth in the research of relationships between small RNAs and chronic pain (Bali and Kuner, 2014). Our RNA-sequencing data displayed above implied the change of small RNAs in the ACC under the condition of NP. To gain a complete picture of miRNAomic signatures in the ACC, small RNA sequencing was further performed in another batch of rats receiving SNI or sham surgery (Supplementary Figure S1).

A total of 11,040,407 clean reads were averagely generated after filtering for low quality from 11,355,792 raw sequencing reads. The number of identified genes and their proportion and distribution to the total gene number in the database of each sample are displayed in Supplementary Table S3. The distribution and density of gene expression levels are shown in Supplementary Figure S3. Most small RNAs were 21-23 nt in length in all the libraries, with 22 nt being the most frequent length, and more than 40.29% were miRNA in the catalog of small RNA in all 14 libraries. A total of 662 mature miRNAs and 165 novel miRNAs were identified in these small RNA libraries.

Concerning miRNA sequencing data analysis, a total of 33 DEmiRNAs were identified, with 31 known and 2 novel miRNAs. Also, 21 and 12 miRNAs were upregulated and downregulated, respectively (Supplementary Figures S4A,B), and a detailed literature review concerning the role of these miRNAs in pain modulation is presented in Supplementary Table S4. Cluster analysis of the DEmiRNAs is shown in Supplementary Figure S4C. Next, we predicted gene targets of 33 DEmiRNAs through the online miRNA targets prediction database to discover the potential miRNA-regulated pathway in NP. A total of 470,667 and 651,166 targeted genes were predicted from the databases RNAhybrid and miRanda, respectively. By intersecting the genes that were predicted in the two databases, we obtained 73,258 predicted genes. GO enrichment and KEGG analyses were finally performed on these genes, and the top 10 terms in BP, CC, and MF and KEGG pathways are shown separately (Supplementary Figure S5). Intriguingly, synapse-related terms and pathways were highly enriched, which was in accordance with the transcriptomic data mentioned above.

### Proteomic signatures in the ACC of rats following SNI

**Figure 5:**
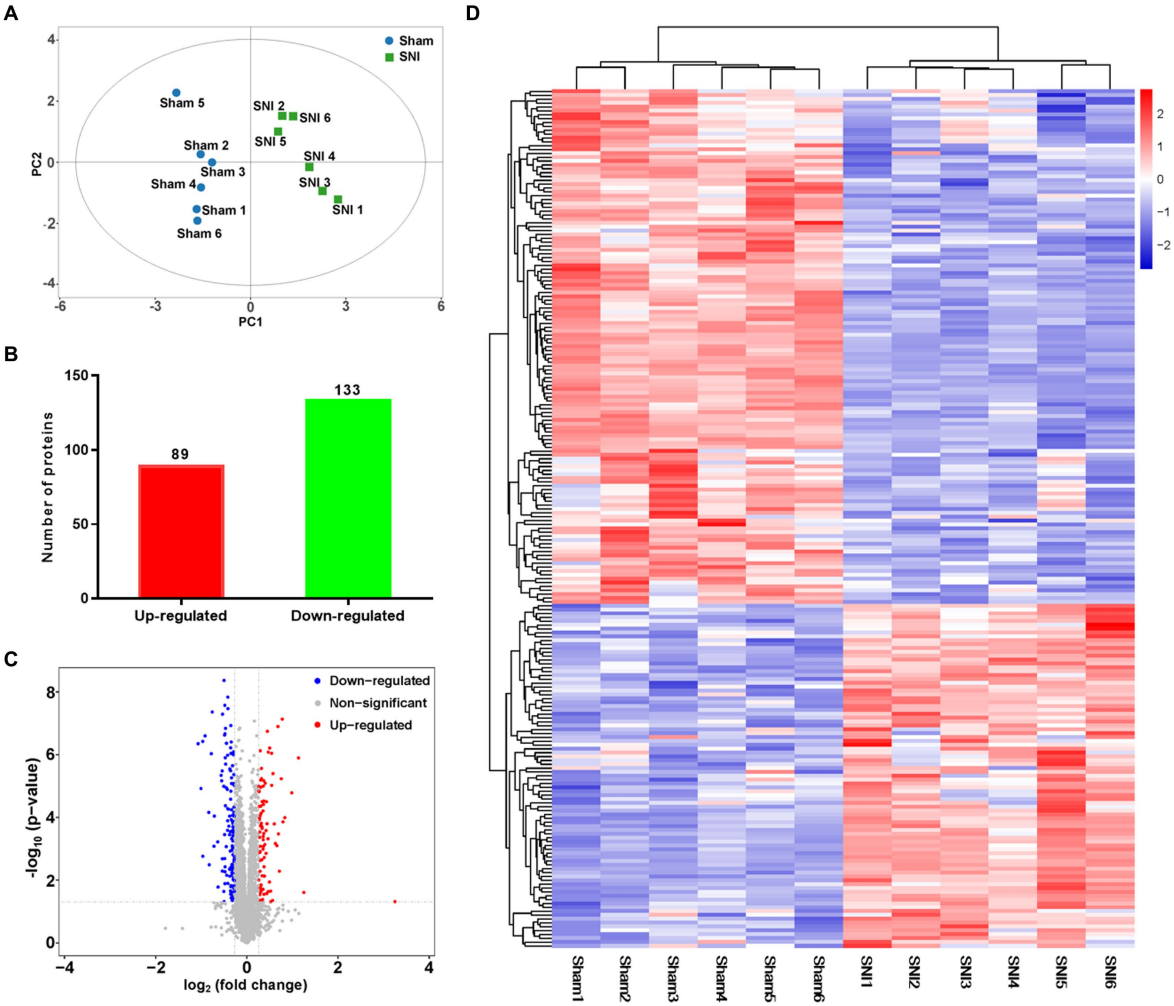
General proteomic outcomes. **(A)** Principal component analysis indicated a near-complete separation of proteins in the sham (blue) and SNI (green) groups. **(B)** In all, 89 proteins were upregulated, and 133 proteins were downregulated. **(C)** Volcano plot shows differentially expressed proteins. The red and blue dots indicate significantly upregulated and downregulated proteins, respectively. **(D)** Hierarchical cluster analysis. Red indicates upregulation, and blue indicates downregulation. The dendrograms represent the classification of proteins. The number in the color scale indicates the z-score.

To quantify the proteomic changes in the ACC of SNI rats, LC-MS/MS was adopted to analyze protein extracts. In total, 6,967 credible proteins were identified, most of which were distributed within the range of 0–200 Da molecular weights (Supplementary Figure S6A) and 0–20 peptide numbers (Supplementary Figure S6B). The results of PCA analysis (Figure 5A) showed that the same group of samples were more concentrated in spatial distribution. Within our identified proteins, we found 89 significantly upregulated and 133 downregulated proteins (Figures 5B,C). The top 20 upregulated/downregulated proteins are shown in Table 2. A heat map further shows the differential expression changes between groups and within each sample of the significantly altered proteins (Figure 5D).

**Table 2:** Differentially expressed proteins in the anterior cingulate cortex after nerve injury by TMT.

Accession	Description	UP	Average		*p*-value	FC
			Sham	SNI		
**Downregulated proteins**						
D3ZF21	GPRIN family member 3 (Gprin3)	12	1189.9	978.3	0.00015	0.82
D3ZCF8	ATP-binding cassette, subfamily A (ABC1), member 8a (Abca8a)	12	1189.0	972.4	0.00528	0.82
P60203	Myelin proteolipid protein (Plp1)	11	9661.4	8010.0	0.04107	0.83
P63036	DnaJ homolog subfamily A member 1 (Dnaja1)	10	1146.8	924.0	0.02215	0.81
B0BND0	Glycerophosphocholine cholinephosphodiesterase ENPP6 (Enpp6)	9	1223.4	910.8	0.00028	0.74
P57113	Maleylacetoacetate isomerase (Gstz1)	8	1146.7	943.2	0.04171	0.82
Q9JKA9	Potassium/sodium hyperpolarization-activated cyclic nucleotide-gated channel 2 (Hcn2)	8	636.5	519.8	0.00639	0.82
D3ZDU5	Profilin (Pfn2)	7	3492.1	2814.2	0.01542	0.81
Q63553	SNF-related serine/threonine-protein kinase (Snrk)	6	260.9	214.6	0.01073	0.82
Q63327	Myelin-associated oligodendrocyte basic protein (Mobp)	6	928.1	733.7	0.01040	0.79
Q811T3	Kv3.3c voltage gated potassium channel subunit splice variant C (Kcnc3)	5	285.1	229.3	0.00033	0.80
Q5XIU4	B-cell receptor-associated protein (Bcap29)	5	465.9	369.8	0.00182	0.79
Q7TP52	Carboxymethylenebutenolidase homolog (Cmbl)	4	278.1	230.0	0.01243	0.83
G3V6B9	Tyrosine-protein phosphatase non-receptor type (Ptpn4)	4	240.5	193.7	0.00501	0.81
M0R4S2	Apolipoprotein D (Apod)	4	273.5	215.8	0.00492	0.79
P07722	Myelin-associated glycoprotein (Mag)	4	1484.1	1103.0	0.00215	0.74
A0A0G2JUM8	Cytochrome c oxidase assembly factor 6 (Coa6)	3	243.0	199.3	0.02272	0.82
G3V8G6	Hyaluronan and proteoglycan link protein 2 (Hapln2)	3	178.2	146.0	0.03783	0.82
D3ZSV7	Similar to THUMP domain containing 3, isoform CRAa (Thumpd3)	3	210.4	169.1	0.00196	0.80
D4A2K5	Nuclear receptor interacting protein 3, isoform CRAa (Nrip3)	3	99.4	76.1	0.01244	0.77
**Upregulated proteins**						
A0A0G2K6S9	Myosin-11 (Myh11)	39	7.4	20.5	0.03966	1.52
C0JPT7	Filamin A (Flna)	39	70.0	169.8	0.02728	1.37
A0A0G2JWK7	Transgelin (Tagln)	11	635.9	1144.1	0.01146	1.56
Q62720	Zinc transporter 1 (Slc30a1)	9	62.0	108.4	0.00035	1.21
Q5FVG5	Similar to tropomyosin 1, embryonic fibroblast-rat, isoform CRAc (Tpm2)	7	19.8	31.8	0.03293	1.80
P07150	Annexin A1 (Anxa1)	6	145.3	229.4	0.03493	1.21
D3ZC96	Tetratricopeptide repeat protein 39B (Ttc39b)	5	891.1	1387.1	0.00768	1.52
A9CMA6	Transmembrane protein 163 (Tmem163)	5	611.2	932.0	0.00054	1.33
A0A0G2K1G8	Solute carrier family 7, member 14 (Slc7a14)	4	2428.6	3697.5	0.01619	1.27
P09330	Ribose-phosphate pyrophosphokinase 2 (Prps2)	4	91.5	134.9	0.00253	1.26
Q64361	Latexin (Lxn)	4	83.2	121.5	0.00746	1.21
D3ZQL1	ER membrane protein complex subunit 7 (Emc7)	4	251.0	362.6	0.04083	1.20
Q923Z2	Tropomyosin 1, alpha, isoform CRA_a (Tpm1)	3	26.1	37.3	0.04930	1.31
Q5U204	Ragulator complex protein LAMTOR3 (Lamtor3)	3	342.7	484.2	0.01251	1.30
Q5BJT2	Ubiquitin-like protein 3 (Ubl3)	3	16.6	23.4	0.00637	1.25
P63255	Cysteine-rich protein 1 (Crip1)	3	222.2	313.5	0.03531	1.24
M0R4L3	PTS1-BP (Pex5)	3	36.8	51.6	0.01220	1.23
Q27W01	RNA-binding protein 8A (Rbm8a)	3	202.9	282.9	0.00407	1.23
D4A4F9	RCG20461 (Stum)	3	162.4	225.8	0.00186	1.23
F1LYA6	Abl-interactor 2 (Abi2)	3	270.6	373.9	0.00209	1.22

Twenty downregulated and upregulated differentially expressed proteins with the lowest *p*-values in the rat anterior cingulate cortex after SNI. The proteins are ranked on the average of respective absolute fold change.

FC, fold change; SNI, spared nerve injury.

**Figure 6:**
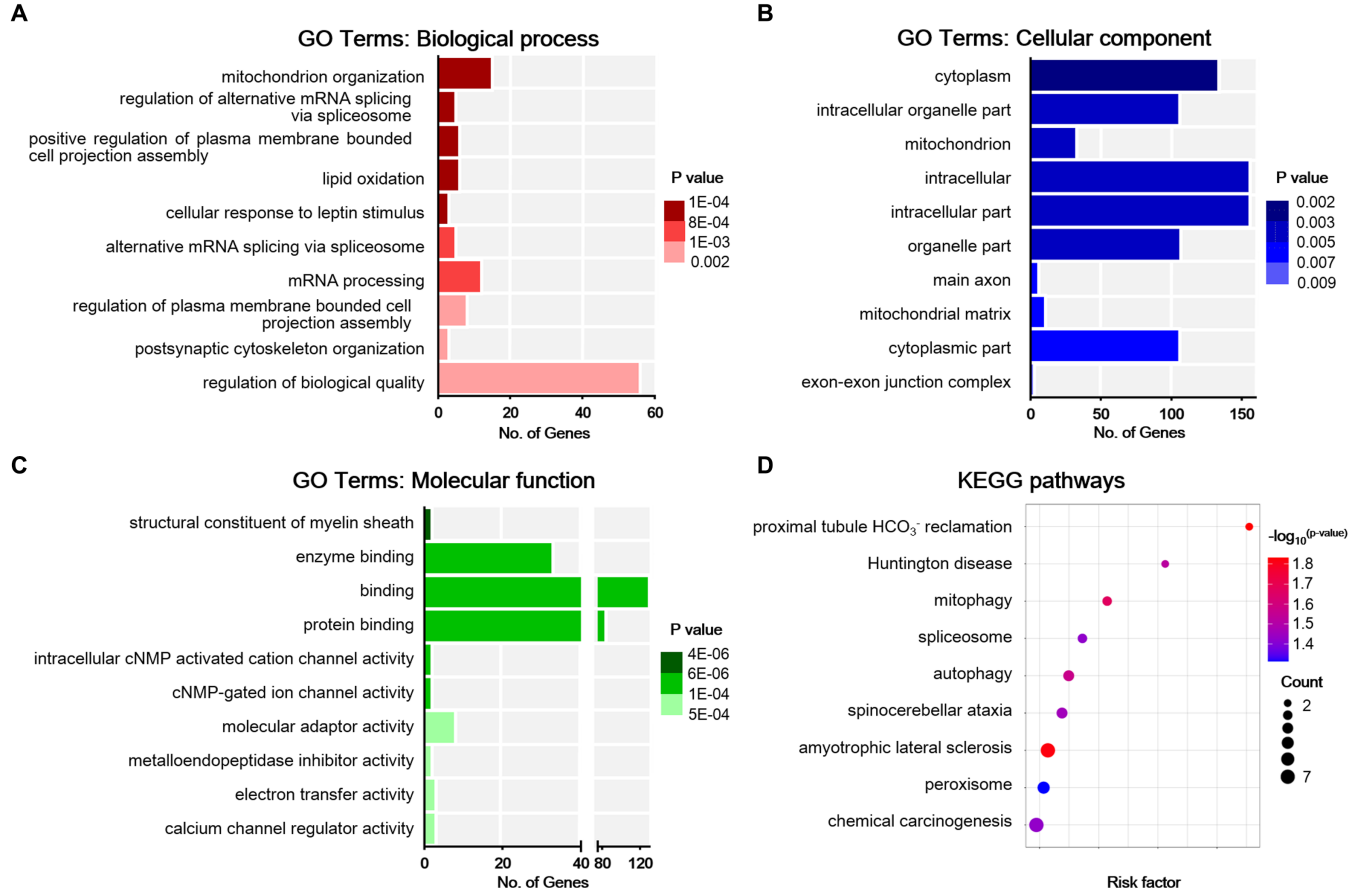
Bioinformatic analysis for differentially expressed proteins in sham and NP rats. **(A)** Biological process. **(B)** Cell components. **(C)** Molecular function. Darker colors indicate higher statistical significance. **(D)** Bubble chart shows KEGG analysis of differential proteins. The horizontal axis represents a rich factor (ratio of the sum of differential proteins enriched in a pathway to the number of proteins annotated by the pathway). Bubble size indicates the number of proteins included in each pathway, and different colors indicate different *p*-values.

To obtain an overview of the effects of SNI on the ACC, GO functional annotation of these 123 DEPs was performed, and the top 10 items according to the *p*-value in each classification are listed in Figures 6A–C. The enrichment results showed that the DEPs were significantly enriched in BPs such as mitochondrion organization, alternative mRNA splicing via spliceosome, plasma membrane bounded cell projection assembly, lipid oxidation, cellular response to leptin stimulus, postsynaptic cytoskeleton organization, and regulation of biological quality. For MF, the DEPs were mainly associated with the structural constituent of the myelin sheath, protein binding, as well as the activity of cyclic nucleotide (cNMP)-gated ion channels, molecular adaptors, metalloendopeptidase inhibitors, electron transfer, and calcium channel regulators. The top CC categories mainly included cytoplasm, intracellular organelle part, and main axon. Through the KEGG database, a total of 10 statistically significant pathways were obtained (Figure 6D). The DEPs were mainly involved in mitophagy, spliceosome, autophagy, and peroxisome. In addition, the KEGG analysis indicated that NP etiology in the ACC shared similar pathways with other neurological diseases, such as amyotrophic lateral sclerosis (ALS), spinocerebellar ataxia (SCA), and Huntington’s disease (HD). These results demonstrated that autophagic and mitochondrial mechanisms are involved in cingulate neurons after peripheral nerve injury.

**Figure 7:**
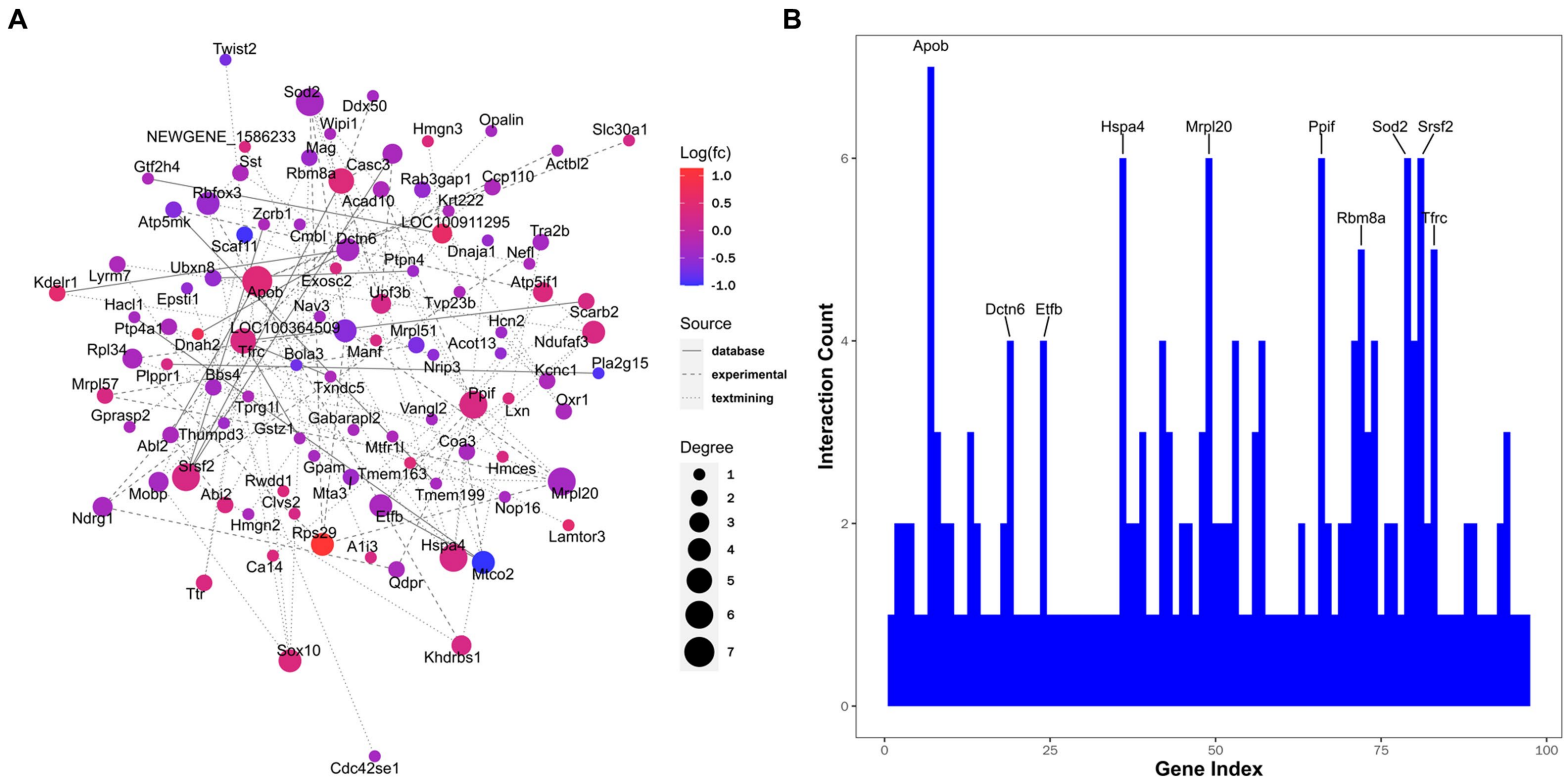
Interaction networks analyzed for differentially expressed proteins. **(A)** Protein-protein interaction. Node color represents the regulation type. Pink nodes indicate upregulated genes and blue nodes indicate downregulated genes. Node size represents the degree value. A larger node size indicates a higher degree value. Different line styles denote different sources of protein-protein interaction. **(B)** Ten proteins with maximum interaction counts are listed.

Finally, the PPI network was constructed, with relevant hub molecules identified (Figure 7A). According to the degree rank, the top 10 molecules were apolipoprotein B-100 (Apob, degree = 7), dynactin subunit 6 (Dctn6, degree = 4), electron transfer flavoprotein subunit beta (Etfb, degree = 4), heat shock 70 kDa protein 4 (Hspa4, degree = 6), mitochondrial ribosomal protein 63 (Mrpl57, degree = 6), peptidyl-prolyl cis-trans isomerase F (Ppif, degree = 6), RNA-binding protein 8A (Rbm8a, degree = 5), superoxide dismutase, mitochondrial (Sod2, degree = 6), serine/arginine-rich splicing factor 2 (Srsf2, degree = 6), and transferrin receptor protein 1 (Tfrc, degree = 5) (Figure 7B).

### Comparison of NP-related changes in mRNA and protein

**Figure 8:**
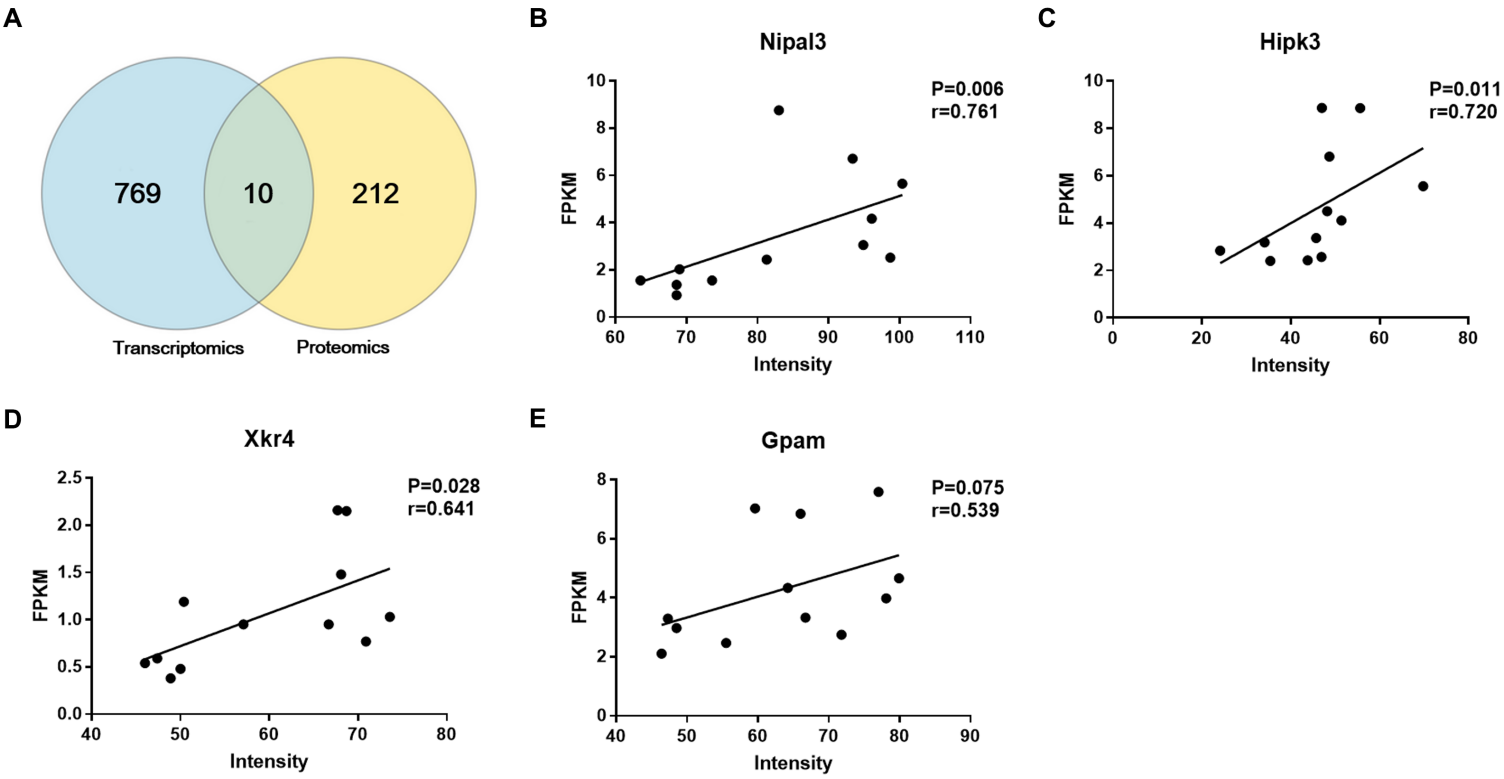
Integrated analysis of transcriptomic and proteomic data. **(A)** Venn diagram showing 10 overlapping targets between transcriptomic and proteomic data. **(B–E)** There is a significant correlation between transcriptomic and proteomic data of Nipal2 **(B)**, Hipk3 **(C)**, and Xkr4 **(D)** instead of Gpam **(E)**. Pearson’s correlation test.

We combined the proteomic and transcriptomic data, and then subjected them to Venn diagram analysis, with 10 overlapping targets finally found (Figure 8A). We looked at concordance in the directions of change between mRNA and protein and identified four genes for mRNA and protein with trends that were concordantly decreasing, including glycerol-3-phosphate acyltransferase 1, mitochondrial (Gpam), XK-related protein 4 (Xkr4), NIPA-like domain-containing 3 (Nipal3), and homeodomain-interacting protein kinase 3 (Hipk3). In addition, we identified six genes with decreasing mRNA and increasing protein levels with NP, including interactor protein for cytohesin exchange factors 1 (Ipcef1), abl-interactor 2 (Abi2), lysosome membrane protein 2 (Scarb2), solute carrier family 7, member 14 (Slc7a14), aquaporin-1 (Aqp1), and neurexin-1-beta (Nrxn1). We further tested the intensity of the association between the proteomic and transcriptomic data of the four targets with consistent trends. Spearman correlation analysis showed strong correlations between mRNA and protein levels in Nipal3 (*r* = 0.761), Hipk3 (*r* = 0.720), and Xkr4 (*r* = 0.641), but no significant correlation in Gpam (*p* = 0.075) (Figures 8B–E).

## Discussion

Although molecular alterations within the ACC after NP have been investigated in prior studies, these data have been limited to RNA-seq of mRNA expression. Thus, the present study utilized a combination of quantitative proteomic and transcriptomic approaches to examine molecular changes within the ACC as a consequence of prolonged exposure to peripheral nerve injury. We were able to confirm previously reported molecular alterations underlying synaptic transmission and plasticity via RNA-seq. However, herein we provided another perspective of understanding through proteomics, indicating the mitochondrial involvement at the cortical level.

### Transcriptomic profiling of the ACC in NP model rats

During the pathological process of NP, noxious signals are transmitted from the periphery to the ACC via the thalamus, which consequently leads to enhancements of cingulate synaptic plasticity and neuronal excitability. LTP of glutamatergic transmission in several pain-related sensory central synapses including the ACC is considered the classic experimental model for studying synaptic mechanisms of chronic pain (Bliss et al., 2016). Our transcriptomic data showed differentially expressed mRNAs enriched in synaptic functions and pathways in the ACC between NP and sham rats, which was subsequently supported by our miRNAome data. Next, we will summarize breakthroughs and insights of these GO terms and KEGG pathways relevant to cingulate synaptic mechanisms under the condition of NP.

Nerve injury triggers excessive glutamate release, which induces Ca^2+^ influx into postsynaptic neurons via NMDAR opening. The expression of NP-related cingulate LTP depends on a complex cascade of Ca^2+^-calmodulin (CaM) dependent signaling pathways. As a pivotal hub of the cascade reactions, adenylyl cyclase 1 (AC1) catalyzes ATP to cyclic AMP (cAMP), which subsequently activates cAMP-dependent protein kinase (PKA). PKA drives the synthesis of several downstream plasticity proteins via phosphorylation of cAMP response element-binding protein (CREB) in the nucleus. These biochemical events ultimately lead to enhanced phosphorylation and membrane insertion of NMDAR and Ca^2+^-permeable AMPAR, thus increasing postsynaptic sensitivity (Xu et al., 2008; Bliss et al., 2016). In addition, AC1 is pivotal for the generation of presynaptic LTP via downstream PKA-dependent signaling (Zhuo, 2016). Inhibiting AC1 activity by selective inhibitors blocks behavioral sensitization and injury-related anxiety in chronic pain models, which is a hopeful strategy for the management of NP (Li et al., 2020). Apart from glutamatergic mechanisms, ACC pathophysiology is featured with cortical disinhibition. Nerve injury facilitates GABA removal from the synaptic cleft, decreases GABA release, and suppresses inhibitory postsynaptic currents in cingulate pyramidal neurons (Narita et al., 2011; Blom et al., 2014). The loss of GABAergic control predisposes hyperactivity of cingulate pyramidal neurons and contributes to pain-related aversion. Thus, upregulating GABAergic inhibitory tone within the ACC provides long-lasting pain relief (Juarez-Salinas et al., 2019).

Our KEGG analysis indicated important roles of cholinergic synapse and estrogen signaling pathways in the ACC under the condition of NP. Accumulating evidence indicated that estrogen in the ACC drove emotional pain via estrogen receptor-β/PKA and G protein-coupled estrogen receptor-1/protein kinase B pathways to promote NMDAR-mediated synaptic plasticity (Xiao et al., 2013; Zang et al., 2020), while cholinergic systems alleviated NP through the activation of GABA_A_R-mediated inhibitory transmission via muscarinic M1 receptors (Koga et al., 2019). In addition to calcium signaling, our GO analysis emphasized the activity of voltage-gated potassium channels (Kv). In 2017, Gao et al. revealed that NP-related cingulate pyramidal neuron hyperactivity was associated with a reduction in Kv2-mediated currents, and functional restoration of Kv2 could reduce neural hyperexcitability and produce analgesia (Gao et al., 2017).

Interestingly, we also noticed the enrichment of adhesion molecules and caveola in the GO terms. Synaptic adhesion molecules play key roles in the modulation of synapse development, neural circuits, and behaviors (Kurshan and Shen, 2019). In accordance with these functions, synaptic adhesion molecules in the ACC have been implicated in a series of brain diseases and dysfunctions (Thomas et al., 2002, 2004). Under the condition of NP, cingulate activation alternates the content of specific adhesion molecules. Among them, neural cell adhesion molecule 1 contributes to behavioral sensitization via spine organization and NMDAR-dependent LTP (Ko et al., 2018; Pan et al., 2018). Caveola, a special type of membrane raft, is implicated in normal physiological processes of the central nervous system (CNS) including endocytosis, cell metabolism, signaling transduction (Allen et al., 2007), spine morphology (Hering et al., 2003), axon guidance (Guirland and Zheng, 2007), and receptor trafficking (Francesconi et al., 2009). As a main scaffolding protein in the caveola, caveolin-1 (Cav-1) plays a key role in synapse formation and plasticity. The dysregulation of Cav-1 is implicated in abnormal neuronal signaling in CNS pathological processes (Gaudreault et al., 2004). Yang et al. first demonstrated that Cav-1 in ACC neurons contributes to NP via promoting membrane insertion of NMDAR (Yang et al., 2015).

### Proteomic profiling of the ACC in NP model rats

Autophagy, an evolutionarily-conserved intracellular process, delivers aged proteins and damaged organelles into the lysosome for degradation. It is pivotal for neuronal survival and synaptic maintenance/plasticity in the CNS and for receptor turnover, synaptic integrity, and myelination in the peripheral nervous system (PNS) (Liu et al., 2019). Pain bears common features with neurodegenerative diseases, such as HD, SCA, and ALS, in which autophagy plays a critical role in the pathology (Deneubourg et al., 2022). As an early event in the origin of NP, elevated autophagic activity in injured nerves is esteemed as an adaptation to stress and to help diminish myelin debris (Marinelli et al., 2014). Although autophagy is differentially regulated in spinal neurons and glial cells following peripheral nerve injury, disrupted autophagic activity is considered to play a key role in the induction and development of NP (Zhang et al., 2013; Liao et al., 2022). In general, autophagic impairment contributes to NP, while autophagic upregulation suppresses pain behavior, possibly by suppressing inflammatory responses (Liao et al., 2022). Correspondingly, drugs aiming at upregulating autophagic levels have been screened out for alleviating NP in preclinical models (Shi et al., 2013; Yin et al., 2018). Emerging data indicated that activating cingulate microglial autophagy ameliorated NP via neuroinflammation suppression (Meng et al., 2020). In accordance with this report, our protein profiling analysis identified the enrichment of significant proteins in autophagic pathways. Thus, autophagy in the cortical pain matrix remains an interesting topic for future studies.

The “mitotoxicity theory” of NP has its origin in chemotherapy-related neuropathy and later proved to be true of peripheral nerve injury. These factors induce mitochondrial dysfunction in primary sensory neurons by disturbing mitochondrial functions (including bioenergetics, transport, fusion, and mitophagy) and enhancing nitro-oxidative stress, which ultimately contributes to axonal growth defects and subsequent sensory neuron sensitization (Bennett et al., 2014; Doyle and Salvemini, 2021). Mitophagy, selective autophagic degradation of damaged mitochondria, constitutes a major mitochondrial quality control pathway. The dysregulation of mitophagy in the PNS leads to aggregation of impaired mitochondria, reactive oxygen species (ROS) production, microglial activation, and myelinoclasis, and as a result, the development of NP (Dai et al., 2020; Huang et al., 2022). At the level of the SDH, impaired mitophagy in microglial cells facilitates NLRP3 inflammasome-mediated neuroinflammation via mitochondrial ROS production, finally promoting pain-related plastic changes in the CNS (Shao et al., 2021). Correspondingly, strategies aiming at improving mitophagy have shown curative effects in experimental NP models (Doyle and Salvemini, 2021; Shao et al., 2021). However, whether mitophagy impairment in supraspinal structures underlines NP remains elusive. Herein, our analysis highlights the key role of mitochondrial dysfunction in the ACC in NP pathophysiology.

Apart from mitochondria, peroxisomes are another type of intracellular organelles maintaining the redox cellular state. Genetic deficit of peroxisome could induce severe demyelination, axonal degeneration, and neuroinflammation (Powers and Moser, 1998; Trompier et al., 2014). Peroxisome involvement has been reported in the development of specific degenerative diseases (Trompier et al., 2014), as well as NP. Chemotherapy-related neuropathy involves peroxisome alterations at the levels of both dorsal root ganglion and SDH (Zanardelli et al., 2014). Correspondingly, peroxisome proliferator-activated receptor (PPAR) agonists emerged as a potential therapeutic strategy (Lan et al., 2007) for NP, owing to their anti-inflammatory and anti-oxidative effects (Freitag and Miller, 2014; Zanardelli et al., 2014). Emerging evidence also hints at the potential role of PPAR signaling in NP suppression via microglial modulation at the level of the ACC (Georgieva et al., 2019). Interestingly, our KEGG analysis further supports the role of cingulate peroxisome in NP pathophysiology.

### Integration of the transcriptomic and proteomic data

Integrative multi-omic analysis revealed several targets that may be involved in NP pathophysiology. Non-imprinted in Prader-Willi/Angelman Syndrome (NIPA)-like domain-containing (NPAL) proteins shares high similarity at the levels of amino acid and domain structure with NIPA proteins that function as transporters or receptors. However, knowledge concerning the function of NPAL is still lacking. So far, there has only been one report showing that the disruption of the function of the mouse Nipal3 gene induced increased IgE Levels, lung dysfunction, as well as behavior deficits (Grzmil et al., 2009). HIPK3 belongs to a family of highly-conserved serine/threonine kinases that mediate developmental processes through the regulation of corresponding transcription factors. It has been reported to participate in apoptosis, inflammation and oxidative stress modulation, steroidogenic gene expression, and glucose metabolism (Rochat-Steiner et al., 2000; Lan et al., 2007; Shojima et al., 2012; Liu et al., 2020). As a phospholipid-scrambling protein in the process of efferocytosis, Xkr4 is specifically expressed in the brain where it, upon activation by caspase 6, induces the exposure of PtdSer on dendrites, axons, and synapses to induce microglial responses for neural network remodeling (Suzuki et al., 2014; Maruoka et al., 2021). Gpam, a key member of the mitochondrial enzyme complex, is involved in the biosynthesis process of triacylglycerol and glycerolphospholipids. Loss-of-function mutation of Gpam has been reported to result in corticospinal tract hypomyelination in patients with cerebral palsy (Li N. et al., 2022).

Although molecular changes in the ACC in the case of NP have been explored in prior studies, these have been limited to RNAseq for mRNA expression (Su et al., 2021; Dai et al., 2022; Zhang et al., 2022). One predisposed assumption of these studies was that changes in mRNA determine changes in gene function (protein levels). However, it should be noted that the transcriptome, especially in the brain, does not often precisely indicate protein abundance (Sharma et al., 2015; Liu et al., 2016). A scientific merit of the present study is that through the combination of proteomic profiling and RNA sequencing, we confirmed priorly reported transcriptional changes in the ACC of NP rats, such as synaptic transmission and plasticity, and also provided another layer of understanding concerning the mitochondrial involvement in NP at the cortical level. Surprisingly, in this study, we observed a low correlation between proteomic and RNA-seq data, with only 10 overlapping targets identified. Even in the overlapping targets, the direction of alteration of a protein under the condition of NP cannot be effectively predicted based on alterations in its coding mRNA. One technical reason for this low correlation is the different sensitivity of these approaches based on the current technical level, with RNA-seq profiles reaching tens of million RNA counts in a regular project, compared to tens of thousands in protein mass spectroscopy tests. Hence, comparing these two sources would unavoidably look at the top fractions of very different collections of reads. Importantly, this phenomenon also suggests that processes driving NP-related alterations at the level of protein may involve changes in rates of mRNA or protein turnover or be post-translation dominated (Vogel and Marcotte, 2012).

Post-translational modifications, in particular fast phosphorylation-based control mechanisms that have not been evaluated in our study, have been linked to advanced brain functions. Synaptic signaling and plasticity have been highlighted as pivotal for the phosphorylation of cytoskeletal, scaffolding, and other synaptic proteins, as well as neuroreceptors (Woolfrey and Dell'Acqua, 2015; Dieterich and Kreutz, 2016). Several excellent reviews have discussed the key role of phosphorylation of synaptic proteins in the ACC under the condition of NP (Qiu et al., 2011; Bliss et al., 2016). Given the above, integration of quantitative phosphoproteomic analysis in the multi-omics is imperative to gain a deeper understanding of molecular substrates of NP. Apart from expression levels, neural activities also change the synthesis and degradation rate of synaptic proteins. Therefore, the interrogation of protein turnover rather than merely expression at the level of synapses has gained increasing interest in the domain of pain research. Recently, Ko et al. identified a series of proteins in the ACC exhibiting changed protein turnover rates but not total levels that contributed to the development of NP via synaptosomal proteome (Ko et al., 2018, 2020). Thus, changes in protein turnover partially account for the low correlation between proteomic and transcriptomic findings identified herein.

## Limitations

First, this study was performed only at a one-time point after SNI. The transition toward a persistent pain condition could be reflected by time-dependent alterations in cerebral protein composition in NP models (Ujcikova et al., 2022). Thus, the dynamics of molecular events after SNI surgery in the ACC remains a topic of future discussion. Second, the study was performed 2 weeks after SNI surgery, when negative pain affections including anxiety have been stably elicited together with behavioral hyperalgesia. Thus, current multi-omic data failed to clarify molecular changes specific to the sensory or emotional aspects of NP. Third, as mentioned above, integrative multi-omics involving phospho-proteomics, metabolomics, and even lipidomics might outline more detailed differences in the molecular landscape for NP. Last but not least, although several potential pathways have been identified herein, functional verification is urgently needed to be performed in the future.

## Conclusion

Overall, these findings revealed that synapse- and mitochondria-related targets in the ACC are potentially involved in NP. Our data provide evidence for understanding the etiology of NP and the theoretical basis for new therapeutic approaches.

## Data availability statement

The datasets presented in this study can be found in online repositories. The names of the repository/repositories and accession number(s) can be found in the article/Supplementary material.

## Ethics statement

The animal study was reviewed and approved by the Ethics Committee of the General Hospital of Norther Theater Command.

## Author contributions

YB, G-BL, and Y-QL designed the study. X-TQ, CG, and L-TM performed the experiments. YB and CG wrote the manuscript. YB, CG, X-NL, F-SH, M-MZ, and Q-YZ performed the data analysis. All authors contributed to the article and approved the submitted version.

## Funding

This research was supported by the National Natural Science Foundation of China (Nos. 82101318 to YB, 81971133 to G-BL, 82130034 and 82221001 to Y-QL), Li Yunqing Expert Workstation of Yunnan Province (No. 202005AF150014 to Y-QL), the Innovation Capability Support Program of Shaanxi (No. 2021TD-57 to Y-QL), and the National Science and Technology Innovation 2030 Major Program (No. 2021ZD0204403 to Y-QL).

## Conflict of interest

The authors declare that the research was conducted in the absence of any commercial or financial relationships that could be construed as a potential conflict of interest.

## Publisher’s note

All claims expressed in this article are solely those of the authors and do not necessarily represent those of their affiliated organizations, or those of the publisher, the editors and the reviewers. Any product that may be evaluated in this article, or claim that may be made by its manufacturer, is not guaranteed or endorsed by the publisher.

## Footnotes

^1^ https://cutadapt.readthedocs.io/en/stable/

^2^ http://www.bioinformatics.babraham.ac.uk/projects/fastqc/

^3^ https://daehwankimlab.github.io/hisat2/

^4^ http://bioconductor.org/packages/release/bioc/html/DESeq2.html

^5^ http://www.geneontology.org

^6^ http://www.genome.jp/kegg

^7^ https://string-db.org/

## Supplementary material

SUPPLEMENTARY FIGURE 1 Systematic workflow of miRNAome analysis in the anterior cingulate cortex of neuropathic pain rat model. **(A)** Schematic diagram shows the key steps of this study. **(B)** miRNAomic workflow.

SUPPLEMENTARY FIGURE 2 The quality of RNA-seq data from each ACC sample. **(A)** Boxplot showing the distribution of gene expression levels in each ACC sample. **(B)** Distribution diagram showing the density of gene expression levels in each ACC sample. **(C)** The constituent ratio of the identified DEGs. RPKM, reads per kilobase million.

SUPPLEMENTARY FIGURE 3 The quality of small RNA sequencing data from each ACC sample. **(A)** Boxplot showing the distribution of gene expression levels in each ACC sample. (B) Distribution diagram showing the density of gene expression levels in each ACC sample. TPM, transcript per million.

SUPPLEMENTARY FIGURE 4 Small RNA sequencing outcomes. **(A)** 21 miRNAs were up-regulated, and 12 miRNAs were down-regulated. **(B)** Volcano plot showed differentially expressed miRNAs. The red and green dots indicate significantly up-regulated and down-regulated miRNAs, respectively. **(C)** Hierarchical cluster analysis. Red indicates up-regulation, and blue indicates down-regulation. The dendrograms represent the classification of miRNAs. The number in the color scale indicates the z-score.

SUPPLEMENTARY FIGURE 5 Bioinformatic analysis for the target genes of differentially expressed miRNAs in sham and NP rats. **(A)** Biological process. **(B)** Cell components. **(C)** Molecular function. Darker colors indicate higher statistical significance. **(D)** Bubble chart shows KEGG analysis of the target genes. The horizontal axis represents a rich factor (ratio of the sum of differential genes enriched in a pathway to the number of genes annotated by the pathway). Bubble size indicates the number of genes included in each pathway, and different colors indicate different p-values.

SUPPLEMENTARY FIGURE 6 Information on the identified proteins in the rats. **(A)** Distribution of the molecular weight corresponding to each qualitative protein. **(B)** Distribution of the peptide number corresponding to each qualitative protein..
