# Supplementary figures and images for: Transcriptomic and proteomic profiling of the anterior cingulate cortex in neuropathic pain model rats
Source: Front Mol Neurosci. 2023 Jun 15;16:1164426. doi: 10.3389/fnmol.2023.1164426 (PMC10311218; doi:10.3389/fnmol.2023.1164426)

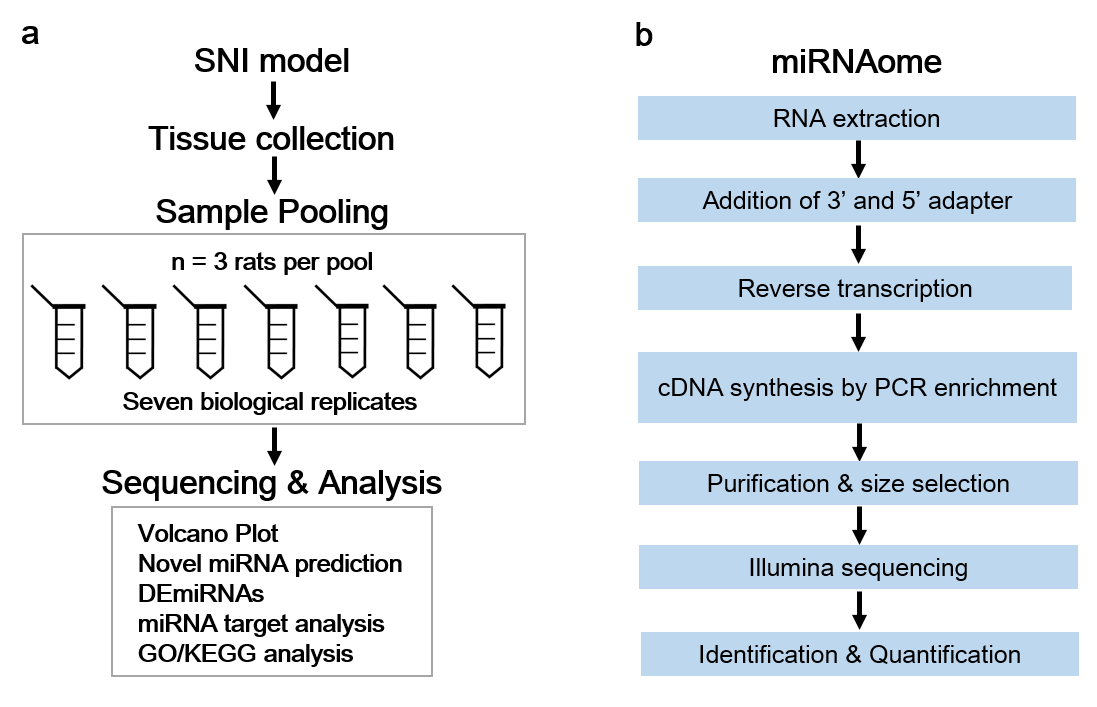

Supplement: SUPPLEMENTARY FIGURE 1 — Systematic workflow of miRNAome analysis in the anterior cingulate cortex of neuropathic pain rat model. (A) Schematic diagram shows the key steps of this study. (B) miRNAomic workflow. [file Image_1.TIF]

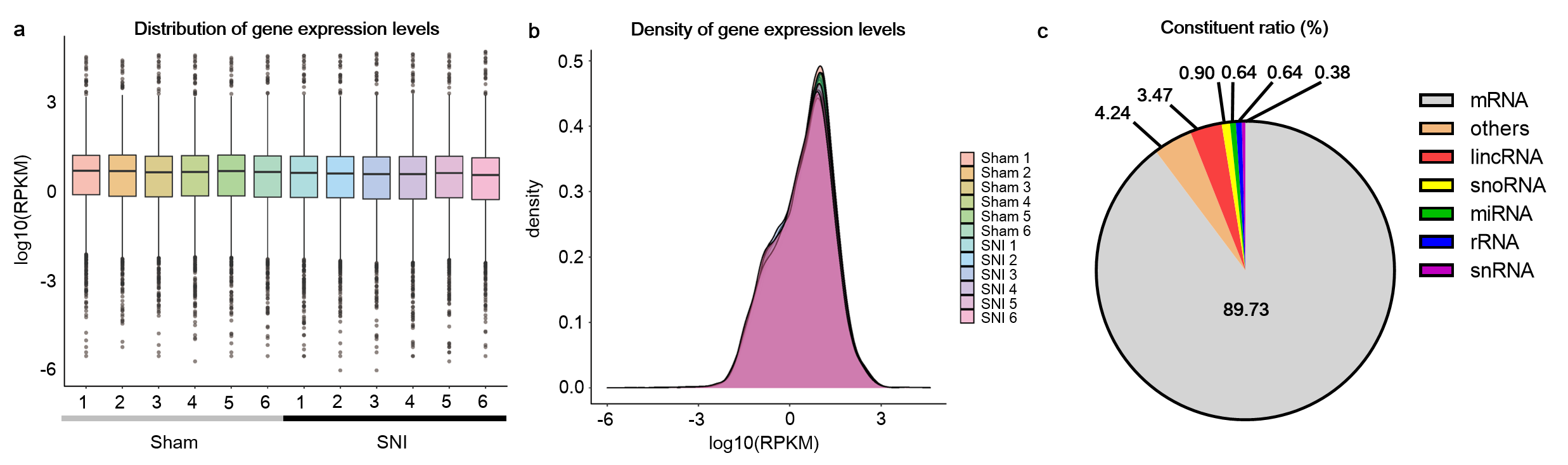

Supplement: SUPPLEMENTARY FIGURE 2 — The quality of RNA-seq data from each ACC sample. (A) Boxplot showing the distribution of gene expression levels in each ACC sample. (B) Distribution diagram showing the density of gene expression levels in each ACC sample. (C) The constituent ratio of the identified DEGs. RPKM, reads per kilobase million. [file Image_2.TIF]

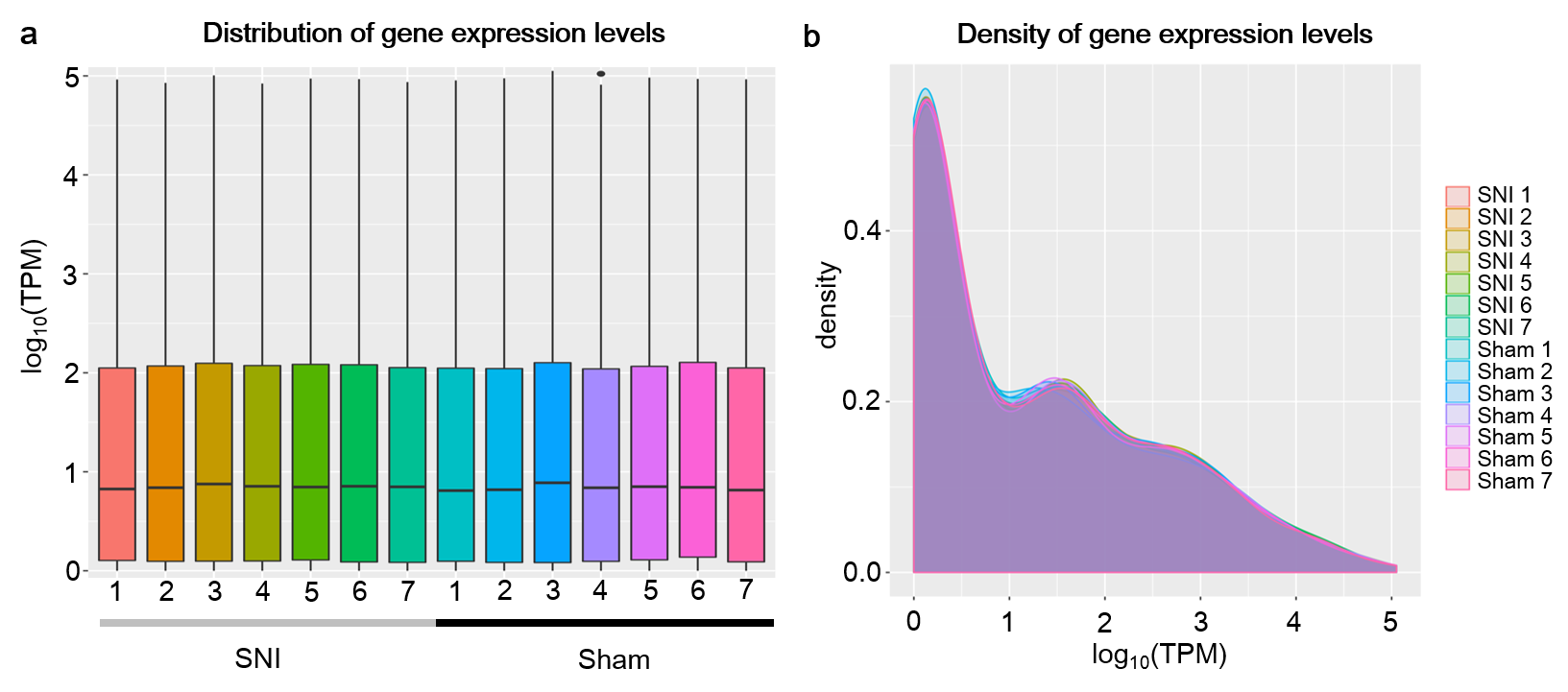

Supplement: SUPPLEMENTARY FIGURE 3 — The quality of small RNA sequencing data from each ACC sample. (A) Boxplot showing the distribution of gene expression levels in each ACC sample. (B) Distribution diagram showing the density of gene expression levels in each ACC sample. TPM, transcript per million. [file Image_3.TIF]

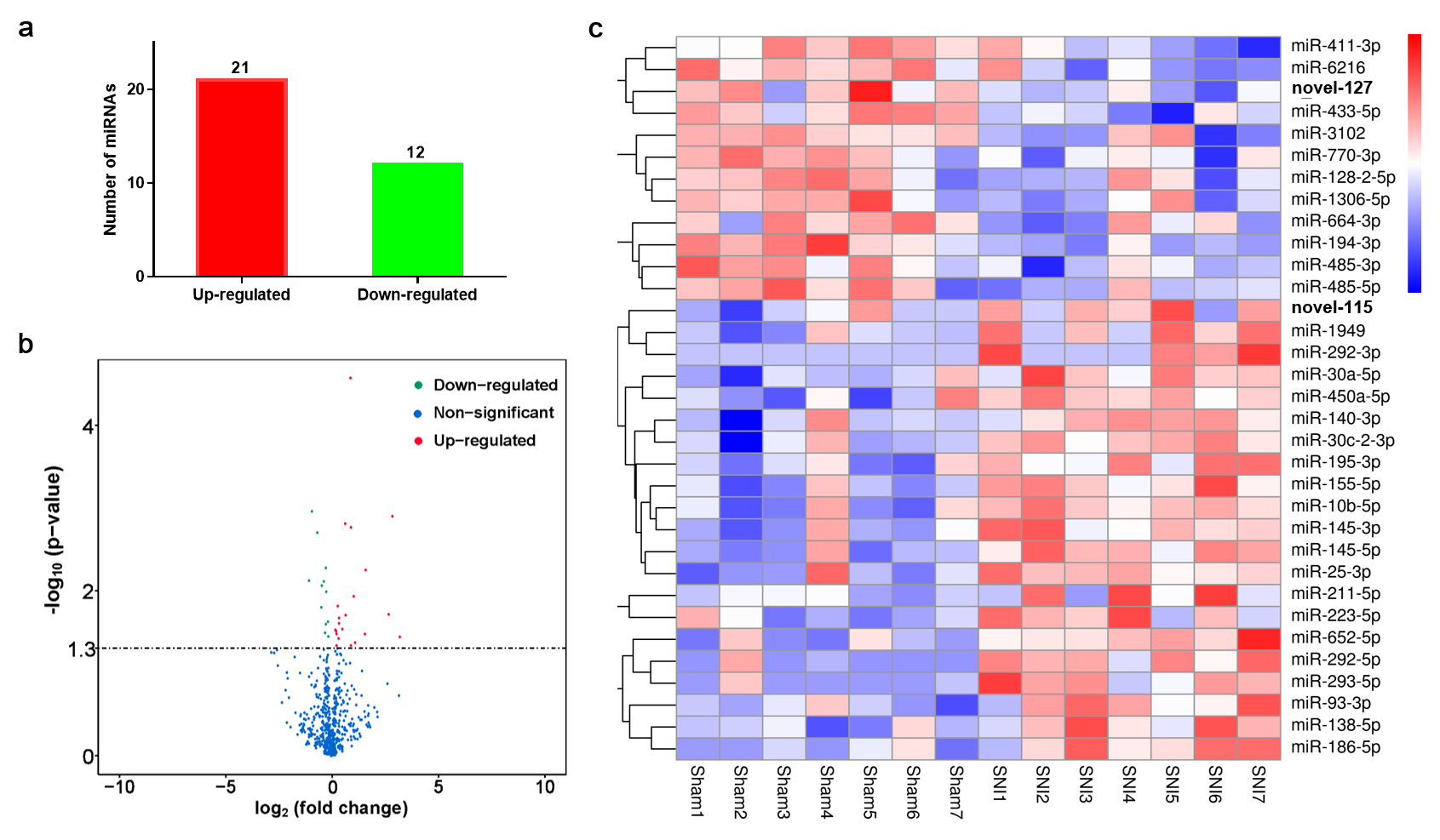

Supplement: SUPPLEMENTARY FIGURE 4 — Small RNA sequencing outcomes. (A) 21 miRNAs were up-regulated, and 12 miRNAs were down-regulated. (B) Volcano plot showed differentially expressed miRNAs. The red and green dots indicate significantly up-regulated and down-regulated miRNAs, respectively. (C) Hierarchical cluster analysis. Red indicates up-regulation, and blue indicates down-regulation. The dendrograms represent the classification of miRNAs. The number in the color scale indicates the z-score. [file Image_4.TIF]

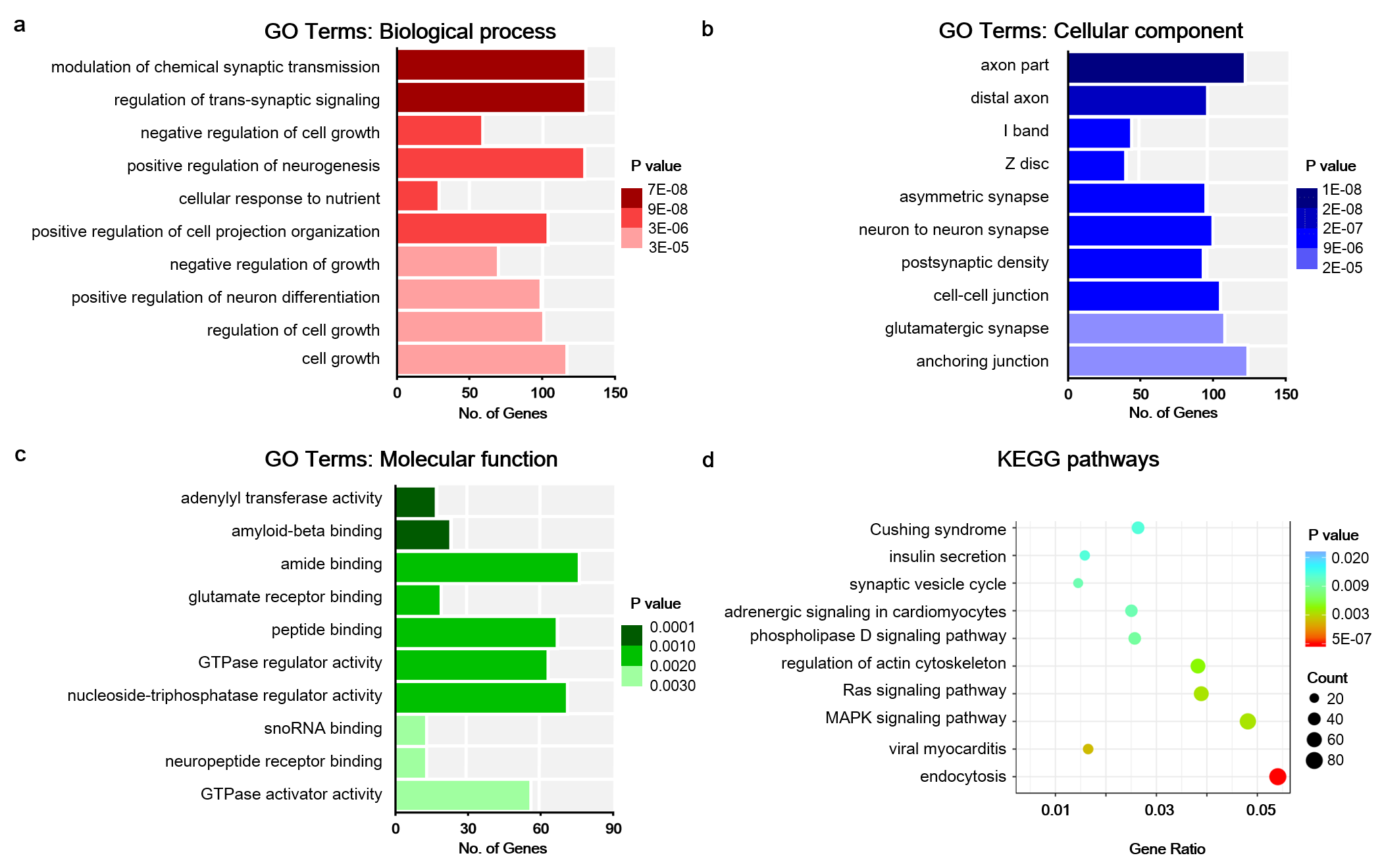

Supplement: SUPPLEMENTARY FIGURE 5 — Bioinformatic analysis for the target genes of differentially expressed miRNAs in sham and NP rats. (A) Biological process. (B) Cell components. (C) Molecular function. Darker colors indicate higher statistical significance. (D) Bubble chart shows KEGG analysis of the target genes. The horizontal axis represents a rich factor (ratio of the sum of differential genes enriched in a pathway to the number of genes annotated by the pathway). Bubble size indicates the number of genes included in each pathway, and different colors indicate different p-values. [file Image_5.TIF]

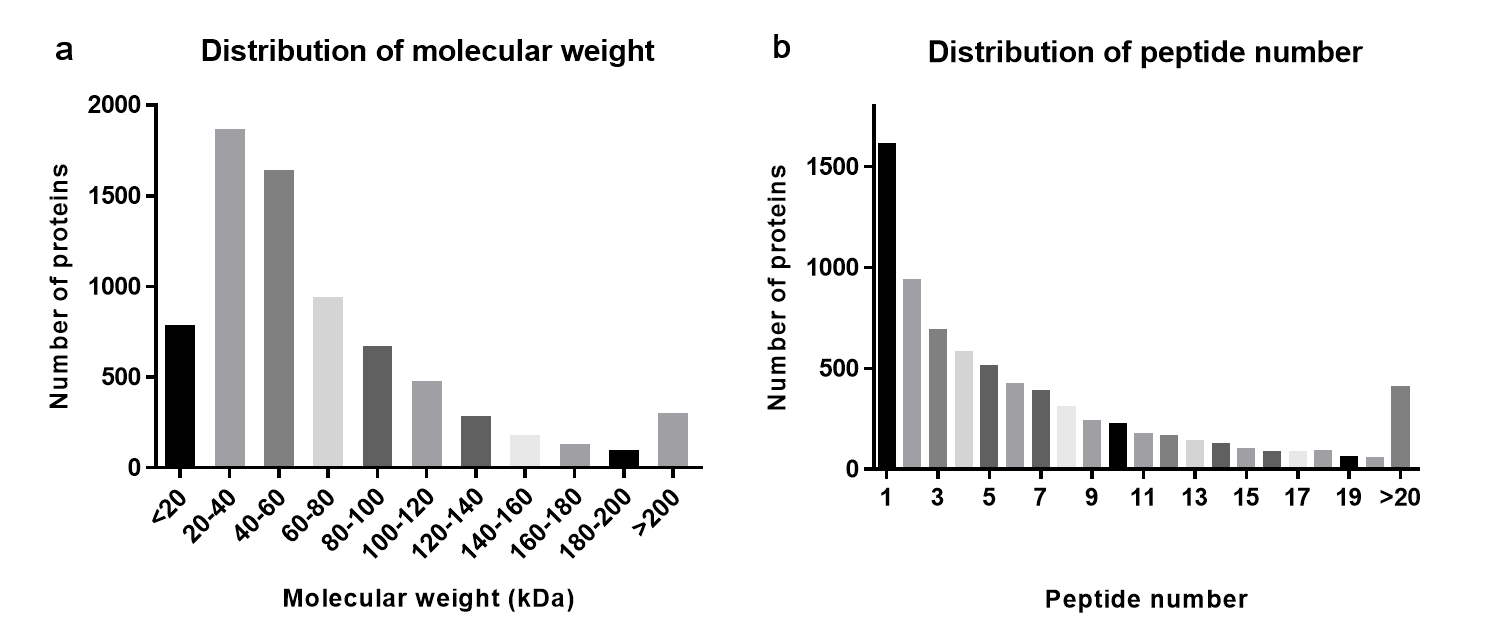

Supplement: SUPPLEMENTARY FIGURE 6 — Information on the identified proteins in the rats. (A) Distribution of the molecular weight corresponding to each qualitative protein. (B) Distribution of the peptide number corresponding to each qualitative protein.. [file Image_6.TIF]
