# Supplementary material for: Transcriptomic and proteomic profiling of the anterior cingulate cortex in neuropathic pain model rats
Source: Front Mol Neurosci. 2023 Jun 15;16:1164426. doi: 10.3389/fnmol.2023.1164426 (PMC10311218; doi:10.3389/fnmol.2023.1164426)
Supplement: Supplementary file 7 [file Table_1.docx]

Supplemental Table 1. The summary of raw general RNA-sequencing data set in RNA sequencing

| Sample | Raw data read | Valid data read | Valid ratio | Q20% | Q30% | GC content% |
| --- | --- | --- | --- | --- | --- | --- |
| Sham 1 | 47484544 | 46087474 | 97.06 | 99.89 | 97.20 | 47.50 |
| Sham 2 | 43275902 | 41960562 | 96.96 | 99.89 | 97.07 | 48.00 |
| Sham 3 | 45300570 | 44021802 | 97.18 | 99.90 | 97.23 | 48.00 |
| Sham 4 | 43094898 | 41795222 | 96.98 | 99.89 | 97.23 | 47.50 |
| Sham 5 | 41120120 | 39731270 | 96.62 | 99.88 | 97.00 | 47.50 |
| Sham 6 | 43404740 | 42131298 | 97.07 | 99.89 | 97.07 | 47.50 |
| SNI 1 | 45404686 | 43945324 | 96.79 | 99.89 | 97.26 | 47.50 |
| SNI 2 | 46303436 | 44902046 | 96.97 | 99.88 | 97.21 | 47.50 |
| SNI 3 | 46432972 | 45008536 | 96.93 | 99.87 | 97.12 | 47.00 |
| SNI 4 | 46771894 | 45207492 | 96.66 | 99.88 | 97.16 | 47.00 |
| SNI 5 | 48185630 | 46731060 | 96.98 | 99.89 | 97.16 | 47.50 |
| SNI 6 | 47999738 | 46482622 | 96.84 | 99.87 | 97.11 | 47.00 |

It showed the summary of RNA-sequencing data of 12 samples, including raw reads number, clean reads

number, valid ratio, Q20 (Phred quality scores Q) and Q30, as well as GC content.

Supplemental Table 2. Differentially expressed non-coding RNAs in the anterior cingulate cortex after nerve injury by RNA-seq

| Gene Name | Description | Average | | p-value | FC |
| --- | --- | --- | --- | --- | --- |
|  |  | Sham | SNI |  |  |
| lincRNA | | | | | |
| AC121220 |  | 0 | 0.22 | 0.00000 | 2221.38 |
| AABR07039000 |  | 2.15 | 5.55 | 0.00000 | 2.58 |
| AABR07044962 | RAN binding protein 2 | 0.91 | 0.28 | 0.00000 | 0.31 |
| AABR07021547 |  | 7.41 | 3.13 | 0.00000 | 0.42 |
| AABR07059004 | RNA binding motif protein 33 | 1.47 | 0.4 | 0.00001 | 0.27 |
| AABR07006097 |  | 0.31 | 0.13 | 0.00004 | 0.44 |
| AABR07032135 |  | 1.89 | 0.8 | 0.00011 | 0.42 |
| AABR07001068 |  | 4.15 | 1.58 | 0.00037 | 0.38 |
| AABR07013729 | afadin- and alpha-actinin-binding protein-like | 0.5 | 0.23 | 0.00043 | 0.46 |
| AABR07069008 |  | 0.58 | 0.28 | 0.00061 | 0.48 |
| AABR07036411 |  | 0.49 | 1.2 | 0.00066 | 2.44 |
| AABR07051326 |  | 1 | 0.42 | 0.00086 | 0.42 |
| LOC102547954 | uncharacterized LOC102547954 | 0.55 | 1.18 | 0.00132 | 2.16 |
| AABR07071395 |  | 1.38 | 0.62 | 0.00157 | 0.45 |
| AABR07008030 |  | 0.93 | 0.34 | 0.00188 | 0.37 |
| AC109886 |  | 0.3 | 0.02 | 0.00232 | 0.05 |
| LOC102550026 | uncharacterized LOC102550026 | 0.5 | 0.24 | 0.00265 | 0.47 |
| AABR07051308 | patatin-like phospholipase domain-containing protein 7-like | 0.32 | 0.14 | 0.00278 | 0.42 |
| AABR07056953 |  | 0.28 | 0.13 | 0.00282 | 0.44 |
| AABR07051879 |  | 1.62 | 0.65 | 0.00638 | 0.40 |
| AABR07041709 |  | 0.12 | 0.01 | 0.00641 | 0.07 |
| LOC102553088 | collagen alpha-1 (XXV) chain-like | 0.76 | 0.34 | 0.00816 | 0.44 |
| AABR07027272 |  | 0.76 | 0.36 | 0.00817 | 0.47 |
| AABR07051882 |  | 1.47 | 0.46 | 0.00838 | 0.32 |
| LOC103690118 | nuclear distribution protein nudE homolog 1-like | 0.22 | 0.04 | 0.00956 | 0.20 |
| AABR07028917 |  | 0.45 | 0.1 | 0.00988 | 0.23 |
| AABR07051882 |  | 1.45 | 0.71 | 0.01023 | 0.49 |
| miRNA | | | | | |
| Mir770 | microRNA 770 | 102.90 | 38.79 | 0.00000 | 0.38 |
| Mir2985 | microRNA 2985 | 18.68 | 8.68 | 0.00048 | 0.46 |
| AC106663 |  | 24.13 | 11.53 | 0.00257 | 0.48 |
| AC135741 |  | 2.93 | 0.99 | 0.00596 | 0.34 |
| AABR07065127 |  | 1.14 | 0.12 | 0.00979 | 0.11 |
| snoRNA |  |  |  |  |  |
| SNORA71 | Small nucleolar RNA SNORA71 | 8.51 | 2.85 | 0.00000 | 0.33 |
| AABR07008715 |  | 2.24 | 5.34 | 0.00050 | 2.38 |
| AABR07006536 |  | 2.27 | 5.48 | 0.00091 | 2.41 |
| AABR07011624 |  | 2.46 | 5.40 | 0.00317 | 2.19 |
| Gm25663 | predicted gene, 25663 | 4.09 | 2.04 | 0.00610 | 0.50 |
| Gm22988 | predicted gene, 22988 | 3.16 | 1.36 | 0.00828 | 0.43 |
| AABR07068327 |  | 0.39 | 1.22 | 0.00870 | 3.15 |
| rRNA |  |  |  |  |  |
| 5_8S_rRNA | 5.8S ribosomal RNA (gene ID: ENSRNOG00000056172) | 1.65 | 4.07 | 0.00069 | 2.46 |
| 5_8S_rRNA | 5.8S ribosomal RNA (gene ID: ENSRNOG00000059394) | 1.72 | 3.72 | 0.00097 | 2.16 |
| 5_8S_rRNA | 5.8S ribosomal RNA (gene ID: ENSRNOG00000058725) | 1.82 | 4.16 | 0.00118 | 2.29 |
| Rn5-8s | 5.8S ribosomal RNA (gene ID: ENSRNOG00000059852) | 1.74 | 3.81 | 0.00241 | 2.19 |
| 5_8S_rRNA | 5.8S ribosomal RNA (gene ID: ENSRNOG00000051831) | 1.30 | 2.75 | 0.00461 | 2.11 |
| snRNA |  |  |  |  |  |
| U2 | U2 spliceosomal RNA (gene ID: ENSRNOG00000054047) | 43.29 | 16.59 | 0.00000 | 0.38 |
| U6 | U6 spliceosomal RNA (gene ID: ENSRNOG00000058615) | 1.38 | 4.56 | 0.00001 | 3.31 |
| U6 | U6 spliceosomal RNA (gene ID: ENSRNOG00000055965) | 1.02 | 0.15 | 0.00711 | 0.14 |

All differentially expressed noncoding RNAs in the rat anterior cingulate cortex after SNI are listed herein.

FC, fold change; SNI, spared nerve injury.

Supplemental Table 3. The summary of raw RNA-sequencing data set in small RNA sequencing

| Sample | Raw data read | Valid data read | Valid ratio | Q20% | Q30% | GC content% |
| --- | --- | --- | --- | --- | --- | --- |
| Sham 1 | 10369194 | 9788532 | 94.40 | 99.33 | 97.59 | 53.16 |
| Sham 2 | 11505095 | 11232712 | 97.63 | 99.31 | 97.55 | 53.03 |
| Sham 3 | 11985984 | 11722854 | 97.80 | 99.37 | 97.78 | 53.78 |
| Sham 4 | 11924032 | 11616313 | 97.42 | 99.33 | 97.64 | 54.24 |
| Sham 5 | 11833231 | 11527451 | 97.42 | 99.35 | 97.72 | 54.20 |
| Sham 6 | 11787670 | 11552073 | 98.00 | 99.31 | 97.6 | 52.93 |
| Sham 7 | 10602970 | 10312521 | 97.26 | 99.35 | 97.73 | 53.05 |
| SNI 1 | 11560550 | 11162185 | 96.55 | 99.34 | 97.69 | 53.53 |
| SNI 2 | 10430915 | 10156296 | 97.37 | 99.23 | 97.34 | 53.10 |
| SNI 3 | 11571211 | 11271265 | 97.41 | 99.36 | 97.70 | 53.75 |
| SNI 4 | 11612543 | 11296234 | 97.28 | 99.22 | 97.38 | 54.10 |
| SNI 5 | 11637320 | 11289827 | 97.01 | 99.37 | 97.82 | 53.84 |
| SNI 6 | 10192912 | 9981985 | 97.93 | 98.70 | 96.16 | 52.30 |
| SNI 7 | 11967464 | 11655446 | 97.39 | 99.33 | 97.62 | 53.26 |

It showed the summary of RNA-sequencing data of 14 samples, including raw reads number, clean reads

number, valid ratio, Q20 and Q30, as well as GC content.

Supplemental Table 4. Differentially expressed miRNAs in the anterior cingulate cortex after nerve injury by small RNA sequencing.

| Gene Name | Potential role in pain modulation | Average | | p-value | FC |
| --- | --- | --- | --- | --- | --- |
|  |  | Sham | SNI |  |  |
| Downregulated miRNAs | | | | | |
| miR-6216 |  | 14.8 | 7.2 | 0.00764 | 0.48 |
| miR-194-3p |  | 22.6 | 11.7 | 0.00110 | 0.52 |
| novel-127 |  | 86.0 | 52.3 | 0.00200 | 0.61 |
| miR-433-5p |  | 40.6 | 28.5 | 0.01608 | 0.70 |
| miR-770-3p |  | 3109.1 | 2251.9 | 0.00870 | 0.72 |
| miR-3102 |  | 151.8 | 115.3 | 0.00778 | 0.76 |
| miR-128-2-5p |  | 948.3 | 755.5 | 0.03255 | 0.80 |
| miR-1306-5p |  | 289.7 | 235.1 | 0.02556 | 0.81 |
| miR-664-3p |  | 1383.0 | 1134.0 | 0.01033 | 0.82 |
| miR-485-3p |  | 4105.8 | 3376.1 | 0.00535 | 0.82 |
| miR-485-5p | Decreased miR-485-5p contributes to inflammatory pain through upregulation of ASIC1 in rat dorsal root ganglion ([Xu et al., 2020](#_ENREF_4)) | 6831.8 | 5929.8 | 0.02399 | 0.87 |
| miR-411-3p |  | 2078.6 | 1860.5 | 0.03631 | 0.89 |
| Upregulated miRNAs | | | | | |
| miR-292-3p |  | 0.2 | 1.6 | 0.03668 | 9.02 |
| miR-293-5p |  | 0.8 | 5.8 | 0.00126 | 7.12 |
| miR-292-5p |  | 2.2 | 13.9 | 0.01939 | 6.41 |
| miR-211-5p |  | 13.7 | 41.1 | 0.00568 | 3.01 |
| miR-223-5p |  | 1.7 | 4.8 | 0.03385 | 2.89 |
| miR-1949 |  | 3.1 | 6.4 | 0.04313 | 2.12 |
| miR-10b-5p |  | 209.2 | 419.7 | 0.01173 | 2.01 |
| miR-155-5p | Spinal miR-155-5p upregulation contributes to mechanical hyperalgesia by promoting microglial activation in bone cancer pain rats  ([He et al., 2022](#_ENREF_1); [Jian et al., 2022](#_ENREF_2)) | 16.6 | 30.6 | 0.00172 | 1.86 |
| miR-652-5p |  | 4.8 | 8.7 | 0.04642 | 1.84 |
| miR-145-5p | Increased circulating miR-145-5p is detected in horses with acute laminitis pain ([Lecchi et al., 2018](#_ENREF_3)) | 5660.2 | 10334.1 | 0.00003 | 1.83 |
| novel-115 |  | 44.4 | 69.1 | 0.01999 | 1.56 |
| miR-145-3p |  | 153.7 | 238.0 | 0.00155 | 1.55 |
| miR-93-3p |  | 25.6 | 35.9 | 0.02945 | 1.41 |
| miR-140-3p | Upregulated miR-140-3p contributes to neonatal repetitive pain via TGF-β3 in rats ([Zhang et al., 2019](#_ENREF_5)) | 13719.3 | 17136.3 | 0.02166 | 1.25 |
| miR-195-3p |  | 140.5 | 174.3 | 0.02504 | 1.24 |
| miR-450a-5p |  | 302.9 | 376.2 | 0.03815 | 1.24 |
| miR-138-5p |  | 1170.2 | 1422.8 | 0.01541 | 1.22 |
| miR-30a-5p |  | 36611.2 | 43088.3 | 0.03120 | 1.18 |
| miR-25-3p |  | 2538.0 | 2945.7 | 0.04645 | 1.16 |
| miR-30c-2-3p |  | 1566.4 | 1801.5 | 0.03394 | 1.15 |
| miR-186-5p |  | 2182.0 | 2499.6 | 0.03009 | 1.15 |

Twelve down-regulated and twenty-one up-regulated differentially expressed miRNAs with the lowest p values in the rat anterior cingulate cortex after SNI. The genes are ranked on the average of respective absolute fold change.

FC, fold change; SNI, spared nerve injury.

References:

He, Q., Liu, L., Wang, Y., Xu, C., Xu, M., Fu, J., et al. (2022). miR-155-5p in the spinal cord regulates hypersensitivity in a rat model of bone cancer pain. *Mol Pain* 18**,** 17448069221127811. doi: 10.1177/17448069221127811.

Jian, Y., Song, Z., Ding, Z., Wang, J., Wang, R., and Hou, X. (2022). Upregulation of Spinal miR-155-5p Contributes to Mechanical Hyperalgesia by Promoting Inflammatory Activation of Microglia in Bone Cancer Pain Rats. *Life (Basel)* 12(9). doi: 10.3390/life12091349.

Lecchi, C., Dalla Costa, E., Lebelt, D., Ferrante, V., Canali, E., Ceciliani, F., et al. (2018). Circulating miR-23b-3p, miR-145-5p and miR-200b-3p are potential biomarkers to monitor acute pain associated with laminitis in horses. *Animal* 12(2)**,** 366-375. doi: 10.1017/s1751731117001525.

Xu, M., Wu, R., Zhang, L., Zhu, H.Y., Xu, G.Y., Qian, W., et al. (2020). Decreased MiR-485-5p Contributes to Inflammatory Pain Through Post-Transcriptional Upregulation of ASIC1 in Rat Dorsal Root Ganglion. *J Pain Res* 13**,** 3013-3022. doi: 10.2147/jpr.s279902.

Zhang, J., Yin, J., Chen, X., Mao, X., Xu, J., Cheng, R., et al. (2019). Down-regulation of miR-140-3p can alleviate neonatal repetitive pain in rats via inhibiting TGF-β3. *Biochem Biophys Res Commun* 515(4)**,** 627-635. doi: 10.1016/j.bbrc.2019.05.133.
